# Exploring the Interplay between Drug Release and Targeting of Lipid-Like Polymer Nanoparticles Loaded with Doxorubicin

**DOI:** 10.3390/molecules26040831

**Published:** 2021-02-05

**Authors:** Tatyana Kovshova, Nadezhda Osipova, Anna Alekseeva, Julia Malinovskaya, Alexey Belov, Andrey Budko, Galina Pavlova, Olga Maksimenko, Shakti Nagpal, Svenja Braner, Harshvardhan Modh, Vadim Balabanyan, Matthias G. Wacker, Svetlana Gelperina

**Affiliations:** 1 Faculty of Medicine, Lomonosov Moscow State University, Leninskiye Gory 1, 119991 Moscow, Russia; kovshova.tatyana.nanofarm@gmail.com (T.K.); j.malinowskaya@gmail.com (J.M.); bal.pharm@mail.ru (V.B.); 2 Faculty of Chemical and Pharmaceutical Technologies and Biomedical Drugs, D. Mendeleev University of Chemical Technology of Russia, Miusskaya pl. 9, 125047 Moscow, Russia; kompacc@yandex.ru (N.O.); kimyaci@list.ru (A.B.); omnews@mail.ru (O.M.); svetlana.gelperina@gmail.com (S.G.); 3 Institute of Human Morphology, Tsurupy 3, 117418 Moscow, Russia; marriott@bk.ru; 4 Institute of Gene Biology, Russian Academy of Sciences, Vavilova 34/5, 119334 Moscow, Russia; lkorochkin@mail.ru; 5 N.N. Blokhin Russian Cancer Research Center, Russian Academy of Medical Science, Kashirskoye Shosse 24, 115478 Moscow, Russia; apbudko@mail.ru; 6 N.N. Burdenko National Medical Research Center of Neurosurgery, 4-ya Tverskaya-Yamskaya 16, 125047 Moscow, Russia; 7 Department of Pharmacy, Faculty of Science, National University of Singapore, 5 Science Drive 2, Singapore 129545, Singapore; shakti.nagpal@u.nus.edu (S.N.); svenja.braner@online.de (S.B.); phahbm@nus.edu.sg (H.M.)

**Keywords:** doxorubicin, PLGA nanoparticles, pharmacokinetics, rats, release kinetics, erythrocyte binding

## Abstract

Targeted delivery of doxorubicin still poses a challenge with regards to the quantities reaching the target site as well as the specificity of the uptake. In the present approach, two colloidal nanocarrier systems, NanoCore-6.4 and NanoCore-7.4, loaded with doxorubicin and characterized by different drug release behaviors were evaluated in vitro and in vivo. The nanoparticles utilize a specific surface design to modulate the lipid corona by attracting blood-borne apolipoproteins involved in the endogenous transport of chylomicrons across the blood–brain barrier. When applying this strategy, the fine balance between drug release and carrier accumulation is responsible for targeted delivery. Drug release experiments in an aqueous medium resulted in a difference in drug release of approximately 20%, while a 10% difference was found in human serum. This difference affected the partitioning of doxorubicin in human blood and was reflected by the outcome of the pharmacokinetic study in rats. For the fast-releasing formulation NanoCore-6.4, the AUC_0→1h_ was significantly lower (2999.1 ng × h/mL) than the one of NanoCore-7.4 (3589.5 ng × h/mL). A compartmental analysis using the physiologically-based nanocarrier biopharmaceutics model indicated a significant difference in the release behavior and targeting capability. A fraction of approximately 7.310–7.615% of NanoCore-7.4 was available for drug targeting, while for NanoCore-6.4 only 5.740–6.057% of the injected doxorubicin was accumulated. Although the targeting capabilities indicate bioequivalent behavior, they provide evidence for the quality-by-design approach followed in formulation development.

## 1. Introduction

For many years, significant attention has been paid to enhancing the selectivity of anti-cancer drugs for solid tumors [1,2]. Clinical trials provide evidence that nanomedicines can optimize pharmacokinetics and tissue distribution, leading to improved therapeutic indices and, sometimes, expanding the therapeutic spectrum. The well-known examples of their clinical success are drug products such as Doxil™, Caelyx™, and Myocet™.

However, targeted delivery of doxorubicin still poses a challenge in cancer therapy [3,4]. More often, the presence of physiological barriers such as the blood–brain barrier (BBB) impacts the specificity of tissue transport as well as the quantities reaching the target site. Although progress has been made in the treatment of brain tumors such as glioblastoma multiforme, a two-year survival rate of less than 25% [5] and a high treatment burden associated with chemotherapy mandate the development of new strategies for drug delivery.

Nanomedicines have been successfully used to deliver biologics [6,7] and small molecular entities to the brain [8,9]. The vast majority of delivery systems employ biomolecules as vectors that facilitate their transport across the BBB [10,11,12]. In parallel, one strategy uses a specific surface modification of nanocarriers that endows it with the ability to attract blood-borne apolipoproteins involved in an endogenous transport of the triglyceride-rich chylomicrons across the BBB [8,13,14,15]. For example, poly(butyl cyanoacrylate) nanoparticles were coated with poloxamer 188, leading to the altered adsorption pattern and providing them with a lipid-like biological identity.

As a consequence, a considerable increase in the survival time of rats with the intracranially implanted 101.8 glioblastoma was observed. Long-term remission was seen in more than 20% of the animals [15]. Apolipoproteins E, B, and A1 were found on the surface of the nanoparticles [8]. The exact pathway of brain uptake was elucidated using nanoparticles covalently modified with apolipoproteins A1 and E [6,16,17]. The vectorized nanocarriers were visualized in the brain using transmission electron microscopy (TEM) [16,17].

Although poly(butyl cyanoacrylate) nanoparticles exhibited a favorable toxicological profile [18], the degradation pathway raised concerns about the toxicity of the material in the brain [3,19]. Only a few years later, a similar effect of the surface coating on brain distribution of polylactide-co-glycolide (PLGA) nanoparticles was confirmed. Poloxamer 188 facilitates the binding of apolipoproteins and induces a similar lipid-like behavior [20]. The early preclinical candidate was characterized by a high anti-tumor efficacy against an intracranially implanted glioblastoma 101.8 in rats [21,22]. It was tested in a phase-I clinical trial in patients with recurrent solid tumors including glioblastoma multiforme. The formulation was well-tolerated without any dose-limiting toxicity even at the highest dose of 90 mg/m^2^ (of doxorubicin) [23].

In the present investigation, we focus on the pharmacokinetics and drug release behavior of these nanoparticles in the blood plasma to estimate the quantities available for drug targeting. Due to the amphiphilic nature of the drug molecule with moderate aqueous solubility, a substantial drug release was expected [24,25]. Following the strategy of active transport of carrier-bound doxorubicin to the brain, this release is responsible for an unparalleled race against time. To elucidate this process, we provide a detailed analysis of the in vivo drug release of two formulation prototypes (NanoCore-6.4 and NanoCore-7.4) and explore the competing forces of drug release and carrier accumulation. In a pharmacokinetic study in rats, we gained more insight into the distribution and elimination behavior of the carrier. To elucidate the role of the encapsulated and the non-encapsulated fraction, the physiologically-based nanocarrier biopharmaceutics (PBNB) model was applied [26]. 

## 2. Results and Discussion

Following the administration of the carrier into blood circulation, several overlapping processes are involved in the distribution and elimination of the drug. The release represents the in vivo conversion between the free and the carrier-bound fraction of doxorubicin. It is a key characteristic that, together with the carrier half-life, is responsible for the capability of nanomedicines to deliver their payload. 

### 2.1. Physicochemical Characterization of Nanoparticles

**Figure 1:**
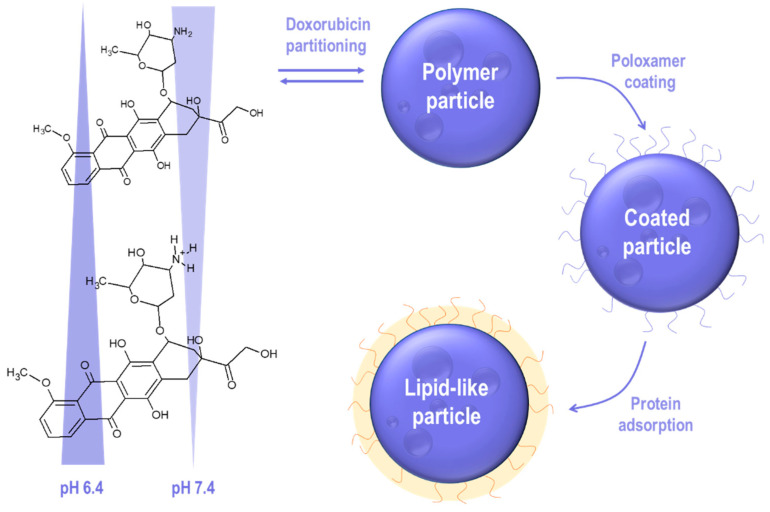
Illustration of the synthesis and adsorption process involved in drug delivery using lipid-like polylactide-co-glycolide (PLGA) nanoparticles loaded with doxorubicin.

To elucidate the role of drug release in the delivery of doxorubicin, two different formulation prototypes exhibiting slight differences in their release behavior were prepared. Both preparations were coated with poloxamer 188 to attract apolipoproteins from blood circulation, leading to a lipid-like biological identity. The amino group in the sugar moiety of doxorubicin enables the solubility of the drug molecule during particle synthesis to be modulated by changing the pH of the aqueous phase. Drug solubility is a key parameter controlling doxorubicin partitioning between the organic and the aqueous phases of the emulsion. The effect of pH on the synthesis of particles as well as the coating procedure resulting in the formation of lipid-like polymer nanoparticles is presented in Figure 1.

At lowered pH values, protonation of the amino group lowers the affinity for the hydrophobic PLGA matrix [27]. Accordingly, the nanoparticle species prepared at different pH values are characterized by different drug load, encapsulation efficiency (Table 1), and release behavior [28].

As expected, NanoCore-6.4 (prepared at pH 6.4) was characterized by a lower encapsulation efficiency of 80 ± 1% as compared to NanoCore-7.4 (prepared at pH 7.4) with an encapsulation efficiency of 91 ± 1%. Before freeze-drying, the nanoparticles exhibited a hydrodynamic diameter of 90–100 nm (Table 2). After freeze-drying of NanoCore-6.4, a fraction of 3–6% of micro-agglomerates (3–5 μm) were found. The average particle diameter of NanoCore-7.4 and NanoCore-6.4 after freeze-drying was 105 ± 12 nm (zeta potential −10.46 ± 1.33 mV) and 137 ± 7 nm (zeta potential −6.40 ± 2.27 mV), respectively.

Coating of PLGA nanoparticles with poloxamer 188 endows them with their lipid-like behavior [29] and enables the successful delivery of doxorubicin to the brain [21,22]. Consequently, the amount of poloxamer 188 adsorbed to the surface of NanoCore-6.4 and NanoCore-7.4 was quantified. A total of 33.65 ± 3.93 μg/mg of the stabilizer was adsorbed. This was in line with previous findings and corresponded to an amount of 0.90 ± 0.08 μg/m^2^ (µg per nanoparticle surface area) [30]. Additionally, this adsorption increased the particle diameter by 11 nm as compared to the particles without surfactant.

### 2.2. Evaluation of the In Vitro Release Kinetics of Doxorubicin

A wide variety of methods has been explored to determine the drug release from nanocarriers including centrifugation [31] and dialysis techniques [25,32]. To gain a better understanding of the mechanisms involved in drug release, different separation methods, and release media were compared. Method optimization is described in the supplementary materials (S1). 

**Figure 2:**
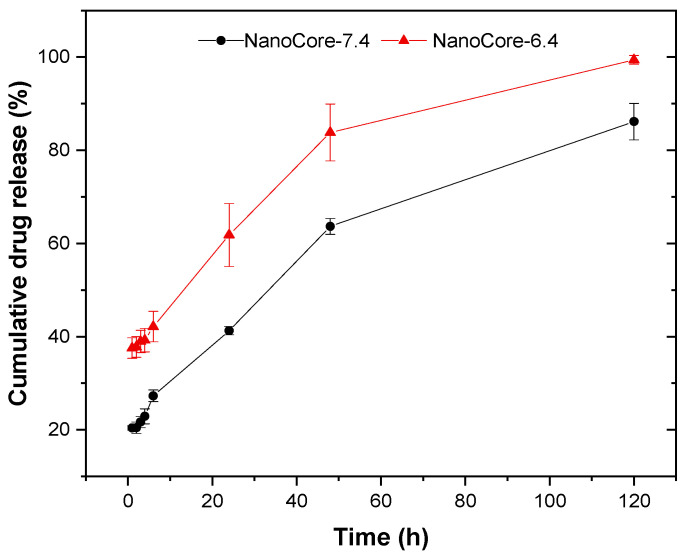
In vitro drug release of doxorubicin from NanoCore-6.4 (red) and NanoCore 7.4 (grey) in an aqueous poloxamer 188 solution (1%) determined by ultracentrifugation at a dilution of 1:25 (*n* = 3, mean ± standard deviation).

In the in vivo situation, the formation of a lipid corona together with a strong dilution of the delivery system is responsible for a comparably high release and increased stability of the colloid system. Therefore, the incubation in an aqueous medium was carried out in presence of a surfactant. NanoCore-6.4 and NanoCore-7.4 were tested in vitro using purified water supplemented with poloxamer 188 (1% solution). The nanoparticle suspension was stable over 120 h. When using an aqueous poloxamer solution as the release medium, the drug release behavior of NanoCore-6.4 and NanoCore-7.4 differed considerably (Figure 2). A pH of 7.4 during synthesis reduces the cumulative drug release by approximately 20% as compared to NanoCore-6.4. Consequently, the two formulation prototypes were suitable candidates to investigate the impact of the bioanalytical method on drug release and pharmacokinetics of particle-bound doxorubicin.

These differences were expected to be less pronounced in the in vivo situation due to the elimination of doxorubicin from the body. The three-dimensional pore structure is a well-known feature of PLGA nanoparticles [33]. Therefore, the adsorption of drug molecules to the particle surface was expected to have a strong influence on the release behavior. To study the influence of diffusion and drug-carrier interactions on the release, different mathematical models were applied [34]. Model parameters were obtained by fitting the in vitro release of doxorubicin using zero and first-order models, as well as the Higuchi, Hickson–Crowell, Korsmeyer–Peppas, and the three-parametric reciprocal powered time (3RPT) model. The outcome of this model fit is described in the supplementary materials (S2). 

During the initial phase of the release, the differences were most significant when calculated with the Korsmeyer–Peppas model. It is often used to describe the release kinetics from the biodegradable polymer matrices (Table 3). The diffusion index n defines the drug release model. For spherical particles, a value of more than 0.43 indicates a release behavior following Fick’s law of diffusion, while an n from 0.43–0.85 corresponds to anomalous transport, and values higher than 0.85 indicate non-Fickian diffusion [35]. Based on this diffusion index, the release was sufficiently explained by Fick’s law of diffusion.

During the first 6 h of the experiment, the diffusion coefficient n was 0.1745 ± 0.0320 for NanoCore-7.4, and 0.0521 ± 0.0078 (*p* < 0.05) for NanoCore-6.4. With regards to NanoCore-6.4, this confirms the weak binding of doxorubicin to the nanoparticles. The rate constant K (Korsmeyer–Peppas model) was 2-fold higher as compared to NanoCore-7.4 (0.3614 ± 0.0057 and 0.1792 ± 0.0367, *p* < 0.05), confirming the differences in the release behavior.

Additionally, the 3RPT model accurately described the release curves. It is applied for the simulation of very different release profiles without providing more information on the exact mechanism. In the context of pharmacokinetic simulation and modeling, it enables the ‘unbiased’ extraction of various release profiles. Following an empirical approach, the model was first developed for solid dispersions [36] and has been applied in a wide variety of biorelevant studies as well [6,25,37]. 

### 2.3. Evaluation of In Vitro Release Kinetics in Human Blood Plasma

The release of doxorubicin from the NanoCore-7.4 and NanoCore-6.4 in human blood plasma was evaluated by determining the free and the total doxorubicin concentrations. The development of the analytical assays used to quantify the drug is described in more detail in the supplementary materials (S3 and S4). 

**Figure 3:**
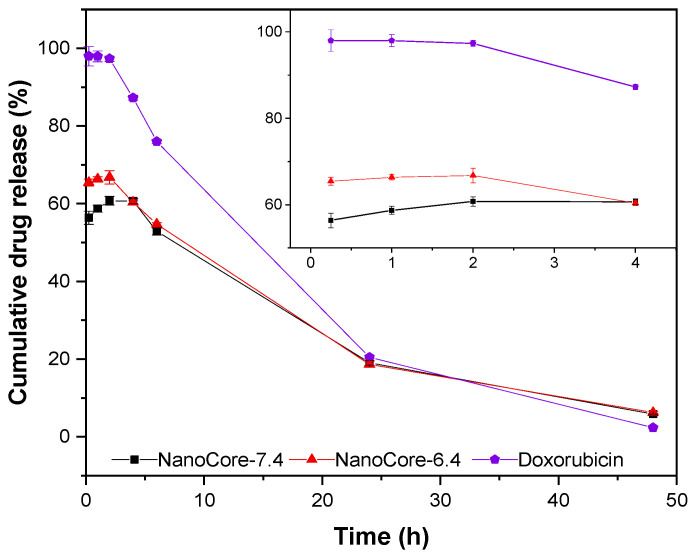
Comparison of in vitro release kinetics of doxorubicin in blood plasma over 48 h and 4 h (inset) from NanoCore-7.4 (grey), NanoCore-6.4 (red) as well as free doxorubicin (blue) as control (*n* = 3, mean ± standard deviation). The release profile is strongly affected by the degradation of doxorubicin in the blood plasma leading to a rapid decrease in the concentration of released doxorubicin.

Importantly, the release profiles obtained under physiologically relevant conditions are strongly affected by the degradation of doxorubicin. At later time points, the concentrations of the reference solution of doxorubicin rapidly decrease [38]. The nanoparticles release approximately 60% of the drug upon dilution (“burst effect”) (Figure 3). The other 40% follow a sustained release behavior. The ratio between release and degradation is responsible for the detected concentration. During the first 4 h, the release from the nanoparticles in the blood plasma followed the same trends that were found for the non-biorelevant medium (Figure 2). However, the release rate was considerably higher, whereas the difference in the total release of doxorubicin from NanoCore-6.4 and NanoCore-7.4 was less pronounced (10%) as compared to non-biorelevant medium (20%). 

As a next step, the interaction between blood cells and the two nanoparticle formulations was investigated in more detail.

### 2.4. Interaction of Doxorubicin and Nanoparticles with Red Blood Cells

The total plasma concentration-time profile provides certain information on the retention of nanoparticles and the drug in the circulatory system. However, the partitioning of the drug between the blood plasma and the blood cells, with the erythrocytes being the main component both in size and in number, may lead to a significant amount being bound to cells and thereby reduce the fraction available for drug targeting.

Consequently, drug partitioning into the red blood cells (RBC) was investigated to enable a more reliable prediction of the pharmacokinetic behavior and biodistribution. Since the interaction with red blood cells was shown for doxorubicin [39] and the PLGA nanoparticles [40], the distribution assay was carried out for the nanoparticulate formulation (free and nanoparticle-bound fraction of doxorubicin) in comparison to the free drug (doxorubicin substance) alone. A quick partitioning of doxorubicin into the erythrocytes was evidenced in rat and human erythrocytes [39,41]. In this study, the assay was performed in whole human blood from healthy volunteers. 

**Figure 4:**
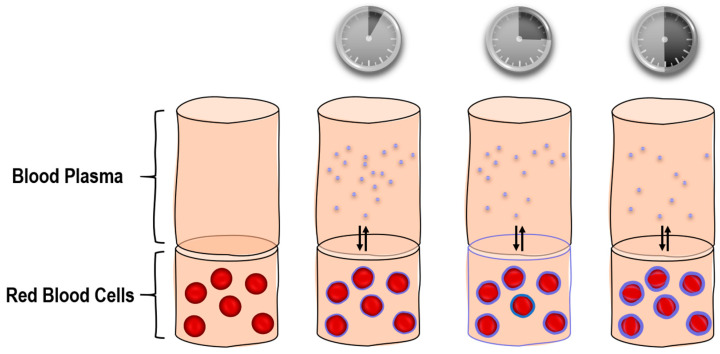
Schematic representation of the distribution assay between the red blood cells and the blood plasma_._

In the present approach, a previously described method [42] was used to determine the blood/plasma and RBC/plasma partition coefficient for the nanoparticulate formulation of doxorubicin at a concentration in a range from 10–100 μg/mL. It provides information on drug partitioning to the erythrocytes without involving a hemolysis step [42], as compared to conventional methods [41,43]. This reduces to overall incubation time [38]. As presented previously, doxorubicin-loaded PLGA nanoparticles do not induce hemolysis for at least 3 h of incubation with whole human blood [28]. An illustration of the assay is presented in Figure 4. 

To quantify the distribution between blood and plasma, the blood-to-plasma rate ratio (*K_Blood/Plasma_*) and the erythrocyte-to-plasma rate ratio (*K_RBC/Plasma_*) of free doxorubicin and the two nanoparticle formulations were determined (supplementary materials, S5). Based on the values of *K_Blood/Plasma_*, the total amount of doxorubicin associated with red blood cells after 5 min of incubation was approximately 33% for both nanoparticle formulations and did not differ significantly from free doxorubicin for the entire concentration range (Table 4).

However, after 15 min of incubation, this fraction increased to 58–63% of doxorubicin for all preparations, 57–58% of NanoCore-6.4, and 46–49% of NanoCore-7.4, and remained at this level until the end of the experiment. As presented in Table 4, both NanoCore-6.4 and NanoCore-7.4 exhibited a lowered affinity for the erythrocytes as compared to free doxorubicin. The difference between the two nanoparticle formulations supports our hypothesis of a considerable fraction of doxorubicin loosely bound to NanoCore-6.4. In general, the nanoparticles can interact with erythrocytes by adsorption to the membrane or by internalization [40]. An equilibrium was reached within a short period.

### 2.5. Analysis of Pharmacokinetics

The plasma-concentration time profiles were collected after intravenous administration of each nanocarrier formulation to adult female Wistar rats. As mentioned above, the coating of the doxorubicin-loaded PLGA nanoparticles with poloxamer 188 essentially contributes to their lipid-like surface pattern and facilitates the delivery into the brain [22]. Accordingly, in this study, NanoCore-6.4 and NanoCore-7.4 were coated with poloxamer 188 before administration to the animals. Key parameters such as c_max_, AUC, Cl, and V were calculated using a non-compartmental approach (Section 2.5.1). In a second step, the PBNB model was applied.

#### 2.5.1. Non-Compartmental Analysis

The outcome of the non-compartmental analysis (NCA) is described in more detail in the supplementary materials (S7). As expected, the nanocarrier formulations led to an increase in c_max_, AUC, and, in particular, AUC_0 → 1h_. The pharmacokinetic parameters of total doxorubicin after administration of NanoCore-7.4 at dose 5 mg/kg were as follows: AUC_all_ was 4603.8 ± 1247.1 ng × h/mL, AUC_0 → 1h_—3589.5 ± 1298.5 ng × h/mL, c_max_—12,441.0 ± 4276.8 ng/mL, Cl—219.5 ± 92.5 mL/h, V—1576.8 ± 789.0 mL. Similar parameters were found after administration of NanoCore-6.4: AUC_all_ was 4434.1 ± 1017.8 ng × h/mL, AUC_0 → 1h_—2999.1 ± 905.1 ng × h/mL, c_max_—10,366.8 ± 2695.4 ng/mL, Cl—216.2 ± 2.4 mL/h, V—2506.4 ± 1242.1 mL. The control group provides strong evidence for rapid elimination of doxorubicin from blood circulation, which correlates with the previous findings [44]. Major drivers are the distribution into lipophilic tissues and an ongoing metabolism and excretion of the drug. The carrier is characterized by a lowered volume of distribution (V) and clearance (Cl).

**Figure 5:**
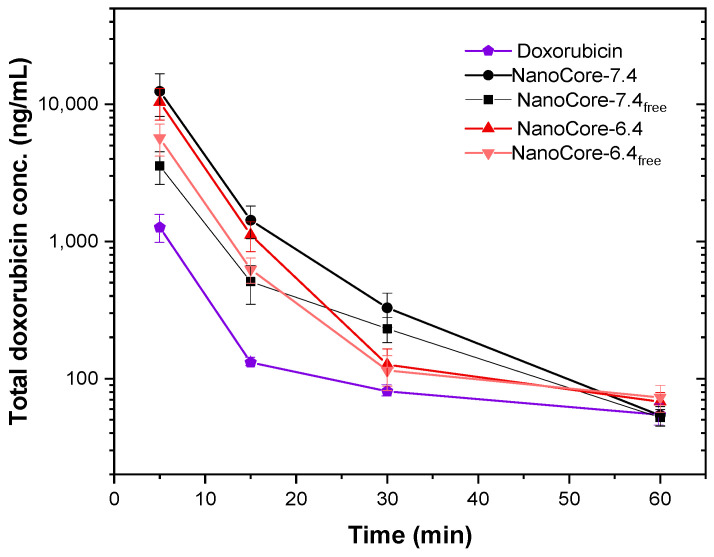
Pharmacokinetic profiles (1 h) for the free and the total doxorubicin concentration following intravenous administration of NanoCore-7.4 (red) and NanoCore-6.4 (grey) in 1% poloxamer 188 as well as a solution of doxorubicin hydrochloride in water (violet) at a dose of 5 mg/kg (*n* = 6, mean ± SD).

Generally speaking, particles with a size of 100–200 nm, exhibit limited mobility in the vascular bed and alter the tissue distribution of the doxorubicin. Over time, they accumulate in the macrophage-rich organs of the reticuloendothelial system (RES). However, after coating the particles with poloxamer 188, the hydrophobic, lipid-like surface is recognized by several receptors leading to enhanced accumulation in the brain [45]. The clearance of doxorubicin bound to the nanoparticles is lower as compared to the free drug. However, this decrease in the plasma concentration is accompanied by a rapid accumulation and, therefore, facilitates targeted delivery (Figure 5). Similar observations were made for other PLGA-based nanotherapeutics [46,47]. 

As compared to the clinical protocols, the t_max_ value corresponds to the first sampling time point with no further delay due to the vascular transit [26]. In the NCA, both formulation prototypes, (NanoCore-6.4 and NanoCore-7.4) are characterized by a very similar AUC_all_ while a considerable difference in the areas during the first hour (AUC_0 → 1h_) indicates a difference in the key characteristics of both carriers. This becomes more evident when looking at the pharmacokinetic profiles of the particles during the first hour. For NanoCore-6.4, total and the free drug concentrations decrease more rapidly compared to NanoCore-7.4 indicating a more rapid release from the nanodelivery system (Figure 5).

To provide a better understanding of the pharmacokinetics, a separate quantitation of the total and the released drug was approached (Figure 5, NanoCore-7.4_FreeDox_, and NanoCore-6.4_FreeDox_). Compared to NanoCore-7.4, the NanoCore-6.4 formulation was characterized with a higher average level of free doxorubicin concentrations at the first minutes after administration: c_max_ values were 3558.2 ± 945.3 ng/mL vs. 5682 ± 1489 ng/mL, respectively. The pharmacokinetic profiles and parameters of the released drug fractions were measured during the first-hour post-injection. They were close to the total drug concentration but differed from one observed for the free drug (Figure 5). 

As presented above, both nanoparticle formulations exhibited a considerable burst effect with approximately 60% of doxorubicin being released within the first hour. This effect is likely due to the difference in drug release observed in vitro between NanoCore-7.4 and NanoCore-6.4. Noteworthy, there is a certain probability for the detection of either protein-bound or particle-bound drug in the free fraction due to the analytical limitations of the method. In the following, the PBNB model was used to further elucidate these differences in more detail and to provide an estimate for the drug release based on the pharmacokinetic data.

#### 2.5.2. Physiologically-Based Nanocarrier Biopharmaceutics Model

**Figure 6:**
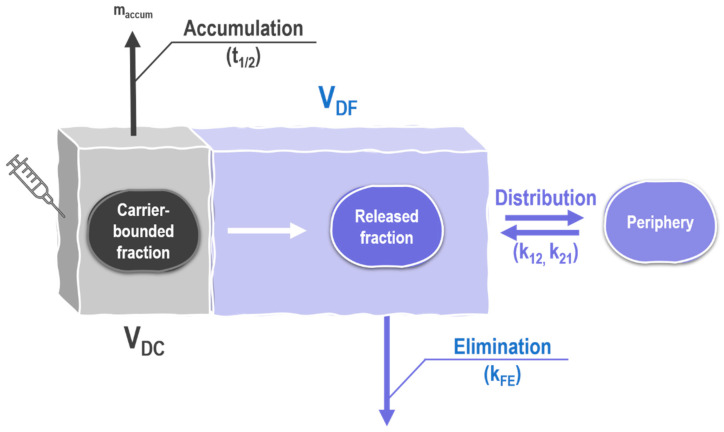
Illustration of the PBNB model. The model is composed of three main compartments representing the carrier-bound (grey) and the released fraction of the drug including the distribution into one periphery compartment (violet).

Compartmental analysis of the plasma concentration-time profiles was carried out using the PBNB model (Figure 6) [26]. It provides a simple but effective alternative to bioanalytical assays that often require a considerable volume of blood. The total plasma-concentration time profile is analyzed based on the model assumption of a volume of distribution of the encapsulated fraction (V_DC_) corresponding to the actual plasma volume of the investigated species [48]. This was confirmed by several investigations and is explained by the limited mobility of large particles in the vascular system [26]. For the present investigation, the physiological blood plasma volume of Wistar rats was used [48]. 

Compared to more conventional modeling approaches [49], the PBNB model uses a very limited number of physiological parameters and summarizes different organs in the accumulation and periphery compartment (Figure 6). This makes it more robust to differences in the exact biodistribution of formulations [26]. The distribution and elimination behavior of free doxorubicin was estimated using the pharmacokinetic parameters observed for the free drug including k_F_, k_12_, k_21_, and V_DF_. 

The model identified the exact volume of distribution of the carrier (V_DC_), the release parameters m, b, and, c as well as the carrier half-life (t_½_) from the total plasma concentration-time curve. The release parameters are based on the 3RPT equation which provides a flexible solution to fit a wide variety of release profiles [50]. Even though this equation is based on Fick’s law of diffusion [36], by introducing a time-dependent variable, it is capable of simulating less predictable influences such as, for example, the formation of a lipid-like corona or the occurrence of a strong “burst effect”.

To provide a scientifically meaningful simulation, the initial estimates play an important role. By narrowing down the range of possible output parameters (t_½_, m, b, c, V_DC_), the number of parameter combinations decreases considerably. Based on our previous investigations we assumed that PLGA nanoparticles rapidly accumulate in different organs [26]. All parameters obtained from this analysis are presented in Table 5.

After initially analyzing the data of both formulations and the two available dose ranges of NanoCore-6.4 (data not shown), a mean half-life of 0.364 ± 0.017 h (ω: 0.5) was applied.

Based on the model assumption that the half-lives may not be strongly influenced by the drug load, this mean value represents a compromise of all fitted training data sets. The volumes of distribution of the carrier (V_DC_) and the targeting capabilities (F_target_) were very similar. The targeting capability of NanoCore-7.4 was ranging from 7.310–7.615%, while the targeting capability of NanoCore-6.4. was ranging from 5.740–6.057%.

**Figure 7:**
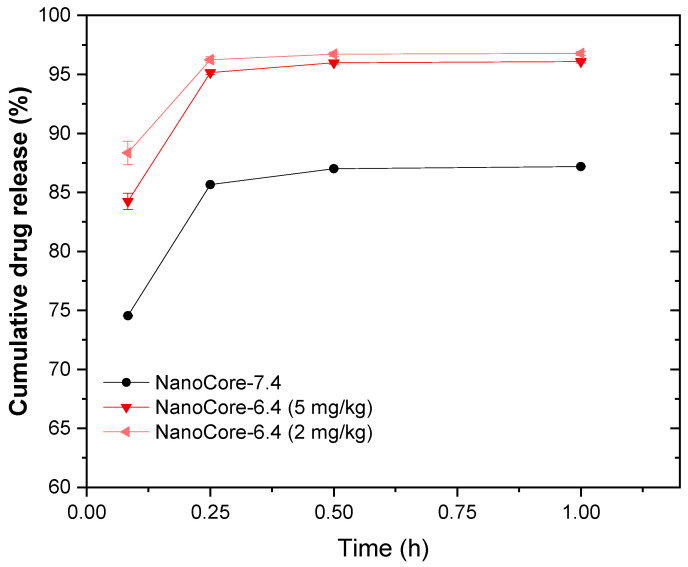
Estimated in vivo drug release (corresponding to the in vivo absorption) using the PBNB model. The error bars indicate the range of simulations obtained with the pre-defined parameter set. They are based on the variability of each simulation parameter used for this calculation.

F_target_ is the fraction of the nanoparticles accumulated in the periphery before the drug is released from the carrier. This finding indicates a very small difference and, considering the variability in pharmacokinetics often seen in animal studies, is likely to be negligible. Therefore, NanoCore-6.4 and NanoCore-7.can be assumed bioequivalent with regards to the overall pharmacokinetics but, more importantly, equivalent with regards to the targeted fraction. The simulated drug release differs by approximately 10% supporting this finding as well (Figure 7).

Still, the influence of the pH during synthesis was reflected by the calculated release curves. The nanoparticles prepared at a pH of 7.4 (NanoCore-7.4) were slightly more efficient as compared to the ones prepared at a pH of 6.4 (NanoCore-6.4). Against this background, the release behavior may not lead to a significant difference in the in vivo performance but still provides a valuable starting point for future design strategies of nanoparticles. Further inhibition of the drug release could lead to more effective delivery systems and lead to more effective treatment of brain cancer.

## 3. Materials and Methods

### 3.1. Materials

Doxorubicin hydrochloride (99.0%) was obtained from Yick-Vic Chemicals & Pharmaceuticals (Hong Kong, China). PLGA (Resomer^®^ 502H, lactide—glycolide ratio of 50:50, carboxylic end groups, Mw 7–17 kDa, η = 0.21 dL/g) was obtained from Evonik Industries AG (Darmstadt, Germany). Polyvinyl alcohol (PVA, 9–10 kDa, hydrolysis degree 80%) and the reference standards daunorubicin hydrochloride (EP CRS, 98.5%, EDQM) and doxorubicin hydrochloride (EP CRS, 99.0%, EDQM) were purchased from Sigma-Aldrich Corporation (St. Louis, MI, USA). Poloxamer 188 (Kolliphor^®^ P188) was obtained from BASF SE (Ludwigshafen, Germany). Acetonitrile (99.9%), orthophosphoric acid (85.0%), and sodium dodecyl sulfate were purchased from PanReac Applichem (Darmstadt, Germany). All other reagents, including dichloromethane and dimethyl sulfoxide (DMSO), were qualified for analytical grade.

### 3.2. Preparation of NanoCore-7.4 and NanoCore-6.4

Doxorubicin-loaded PLGA nanoparticles were synthesized by using the double emulsion solvent evaporation technique (w/o/w). In brief, an amount of 120 mg of doxorubicin hydrochloride was dissolved in 4.8 mL of hydrochloric acid (0.001 N) and added to a solution of 1.2 g of PLGA in 7.2 mL of dichloromethane. The mixture was emulsified using an Ultra-Turrax T18 Basic high shear rotor-stator mixer (IKA Industrie- und Kraftfahrzeugausrüstung GmbH, Königswinter, Germany) for 1 min at 23,600 rpm. The pre-emulsions were added to a volume of 60 mL of an aqueous solution of PVA (1%) in phosphate-buffered saline (PBS) at pH 7.4 (to obtain NanoCore-7.4) or pH 6.4–6.5 (to obtain NanoCore-6.4). The mixture was further emulsified using the high shear rotor-stator mixer (Ultra-Turrax T-18) over 2 min. To further reduce the particle size, the emulsion was passed through a high-pressure homogenizer (Microfluidizer M-110P, Microfluidics, Newton, MA, USA) at 15,000 psi for 3 min while maintaining a temperature of +20 °C. The remaining organic solvent was removed under vacuum (20 mbar) using a rotary evaporator (Laborota 4000, Heidolph Instruments GmbH and Co. KG, Schwabach, Germany). The suspension was passed through a glass porous filter (pore size 90–150 µm). A total amount of 5% (*w*/*v*) of mannitol was added as a cryoprotectant. The dispersions were filled into freeze-drying vials (1.5 mL per vial) and freeze-dried using an Alpha 2–4 LSCplus free dryer (Martin Christ GmbH, Osterrode, Germany). The freeze-dried particles were stored at +4 °C. 

To modify the surface of the nanoparticles, the freeze-dried nanoparticles were resuspended in the aqueous solution of poloxamer 188 (1%) incubated for 30 min before further characterization.

### 3.3. Characterization of Formulation Prototypes

Initially, the physicochemical characteristics of each formulation were determined. This included the particle size, size distribution, zeta potential as well as the drug load, the encapsulation efficiency, the contents of PLGA and poloxamer.

#### 3.3.1. Measurement of Nanoparticle Size, Size Distribution, and Zeta-Potential

The particle diameter and polydispersity index (PDI) were determined by dynamic light scattering using a Zetasizer Nano ZS (Malvern Instruments, Malvern, UK). The ζ-potential was determined by laser Doppler microelectrophoresis in a Malvern dip cell. All measurements were performed after dilution of the suspension to a polymer concentration of 200 μg/mL.

#### 3.3.2. Evaluation of Drug Content and Encapsulation Efficiency

The formulations were characterized for the total and the encapsulated drug content using a centrifugation method. The total content of doxorubicin was determined spectrophotometrically at a wavelength of 481 nm after the dissolution of freeze-dried nanoparticles in DMSO. Linearity was determined in a concentration range of 0.00 to 50.0 μg/mL (R^2^ = 0.9995). The concentration of free doxorubicin was obtained after separation of the nanoparticles by ultracentrifugation at (48,254× *g*, 30 min, +5 °C) using an Avanti JXN-30 Centrifuge System (Beckman Coulter, Pasadena, CA, USA). Linearity was determined in a concentration range of 0.00 to 52.5 μg/mL (R^2^ = 0.99809). The encapsulation efficiency of doxorubicin (*EE*, %) was calculated as the ratio (%) of the difference between total and free doxorubicin to its total content in the sample (Equation (1)):

(1) $$EE\left(\%\right)=\frac{C_{total}-C_{free}}{C_{total}}\times 100\%$$

where *c_total_* (μg/mL) represents the total doxorubicin concentration in the sample and *c_free_* (μg/mL) the concentration of free doxorubicin. All measurements were performed in triplicates.

#### 3.3.3. Evaluation of PLGA Content

The total PLGA content was determined by capillary zone electrophoresis as described previously [51]. In summary, a CAPEL-105M capillary electrophoresis system equipped with a spectrophotometric detector and a quartz capillary tube (internal diameter 75 µm, effective length 50 cm, total length 60 cm) in combination with Elforun^®^ software were used (Lumex Industries, Mission, Canada). A volume of 2 mL of 0.1 sodium hydroxide solution was added to each vial with freeze-dried nanoparticles and incubated at +37 °C under constant stirring (200 rpm, 24 h). Afterward, the hydrolysate was diluted 1000-fold with water before analysis. The concentration of lactic acid monomers was measured at 254 nm (detection time—4 min). The carrier electrolyte solution comprised 10 mM benzoic acid, 0.5 mM cetyltrimethylammonium hydroxide and the pH was adjusted to 8.6 using 9 mM solution of diethanolamine. The amount of polymer was determined by the peak of lactic acid on the electropherogram using a calibration curve. The drug loading was calculated following Equation (2):

(2) $$DL\left(\%\right)=\frac{m_{Doxorubicin\;\left(total\right)}}{m_{PLGA\left(total\right)}}\times EE\left(\%\right)$$

where *DL*(%) represents the total drug load, *m_Doxorubicin (total)_*—the total content of doxorubicin in the formulation, and *m_PLGA (total)_*—the total content of PLGA.

#### 3.3.4. Evaluation of Poloxamer 188 Content

Poloxamer 188 was used to modify the surface of the nanoparticles and is responsible for the modulation of the protein corona. Consequently, the amount of poloxamer adsorbed to the nanoparticle surface was quantified. Each formulation was analyzed using a modified iodometric method as described previously [30]. It is based on the interaction of poloxamer 188 with iodine leading to the formation of colored compounds with a characteristic absorption maximum (λ_ex_ 501 nm) [52]. The poloxamer content was calculated from the difference between the poloxamer concentrations of the surfactant solution before and after incubation of the nanoparticles and expressed relative to the surface area of the nanoparticles (µg/m^2^). 

### 3.4. In Vitro Release Studies Using Simplified Release Media

To quantify the release rate a broad array of methods and media were evaluated. The optimization of the method and justification of the selected conditions are described in supplementary materials (S1). As a result, the in vitro release of doxorubicin from the nanoparticles was evaluated in water supplemented with 1% (*v*/*v*) of poloxamer 188 using a centrifugation method under different conditions. Initially, the freeze-dried nanoparticles were resuspended in the release medium and further diluted to a doxorubicin concentration of approximately 68 µg/mL and a final volume of 25 mL (25-fold dilution). The incubation was carried out at a temperature of +37 °C under permanent shaking. Additionally, the kinetics of release was studied using a 5-fold dilution of the suspension (at a doxorubicin concentration of approximately 340 µg/mL). At pre-determined time points (1, 2, 3, 4, 6, 24, 48, and 120 h), samples with a volume of 1.5 mL were collected and the nanoparticles were separated from the release medium by centrifugation (48,254× *g*, 30 min) at a temperature of +5 °C in an Avanti JXN-30 centrifuge (Beckman Coulter, Pasadena, CA, USA). For comparison, centrifugation at a lower speed was evaluated (15,000× *g*). The concentration of doxorubicin in the supernatant was measured spectrophotometrically at a wavelength of 481 nm Linearity was determined in a concentration range of 0.00 to 59.9 μg/mL (R^2^ = 0.9994). The percentage of free doxorubicin was calculated as follows:

(3) $$F=\frac{M_{t}}{M_{\infty }}\times 100\%$$

where *M_t_* is the amount of doxorubicin in the supernatant at a given time point while *M*_∞_ is the total amount of doxorubicin.

### 3.5. In Vitro Release Studies Using Human Plasma

Human blood plasma was a generous gift of the N.N. Burdenko National Medical Research Center of Neurosurgery (Moscow, Russia). The experiment was approved by the ethics committee of this institution.

For separation of the nanoparticles from the free fraction of the drug, a centrifugation method was used. The freeze-dried nanoparticles were resuspended in purified water and further diluted with plasma to a doxorubicin concentration of 68 µg/mL and final volume of 25 mL, followed by incubation at +37 °C under continuous shaking. At predetermined time points (15 min, 1, 2, 4, 6, 24, and 48 h), the 1.5 mL aliquots were collected, and the nanoparticles were separated by centrifugation (48,254× *g*, +5 °C, 30 min). Instead of the doxorubicin-loaded PLGA nanoparticles, a solution of doxorubicin hydrochloride in an appropriate concentration was used as control. 

The percentage of free doxorubicin (release fraction) was calculated relative to the initial total content of doxorubicin (Equation (3)). The drug content (free and total) in plasma were quantified using high-performance liquid chromatography (HPLC). Before the injection of the samples, the drug was extracted using a mixture of DMSO and acetonitrile (1:1) supplemented with 0.1% of formic acid. Different extraction methods and different centrifugation regimens for nanoparticle separation were evaluated before this investigation (Supplementary material S3, S4). 

Daunorubicin (EP CRS, 98.5%, EDQM), which is close to doxorubicin in terms of the extraction and chromatographic parameters, was used as the internal standard for the quantification method. Sample preparation and the preparation of stock solutions are described in more detail in the Supplementary materials S3.

### 3.6. Quantification of Doxorubicin in Human Plasma

For quantification of doxorubicin (free and total) in human plasma, an HPLC method was used. The reversed-phase HPLC assay was performed at isocratic elution mode using a Shimadzu System (Kyoto, Japan) equipped with a Waters (Milford, CT, USA) Symmetry column (3.9 × 150 mm, particle size 5 μm). A spectrophotometric detector was used at a wavelength of 254 nm. The mobile phase was composed of 2.88 g/L sodium dodecyl sulfate in 2.25 g/L in orthophosphoric acid and acetonitrile (51:49) delivered at a rate of 1.0 mL/min. The column temperature was set to 25 °C. The limit of quantitation was 1.95 µg/mL. For quantification of the drug from plasma samples, the coefficient of variation of precision and accuracy was less than 15% and recovery was in the range of 96–104%. The concentrations of doxorubicin were calculated according to a calibration curve with normalization to the internal standard (daunorubicin) according to Equation (4):

(4) $$\frac{S_{doxorubicin}}{S_{IS}}=0.0628\times c_{doxorubicin}+0.0219$$

where *S_Doxorubicin_* and *S_IS_* represent the peak areas of doxorubicin and the internal standard, $c_{doxorubicin}$ represents the doxorubicin concentration [µg/mL] (R^2^ = 0.9999). All measurements were performed in triplicates.

### 3.7. Analysis of In Vitro Drug Release Using Different Mathematical Models

The in vitro drug release profiles were analyzed using different mathematical models describing the kinetics of drug release from polymer matrices. The models applied to the in vitro release data are summarized in Table 6. 

For the analysis, the release curves were divided into two sections including the rapid release phase (1–6 h) and the continuous release phase (6–120 h). The curve was analyzed for the entire time of doxorubicin release (1–120 h). To compare the kinetics of doxorubicin release from the doxorubicin nanoparticles, different release models were evaluated and a comparison of 48-h doxorubicin release profiles was performed. 

### 3.8. Interaction of Nanoparticles with Human Red Blood Cells

Human blood was a generous gift of the N.N. Burdenko National Medical Research Center of Neurosurgery (Moscow, Russia). The experiment was approved by the ethics committee of this institution. The ratios of the total doxorubicin concentration in whole blood to the blood plasma concentration (*K_Blood/Plasma_*) and the total doxorubicin concentration in red blood cells (RBC) to the plasma concentration (*K_RBC/Plasma_*) provide an estimate of the drug amount bound to the cellular fraction of the blood. The method to determine these parameters was adapted from Yu et al. [42]. In this case, *K_Blood/Plasma_* is the ratio of total doxorubicin concentration in the control plasma (*c_cp_*) to the total doxorubicin concentration (*c_p_*) in the b-plasma obtained after separation of red blood cells by centrifugation of whole blood. In a first approximation, *c_cp_* should equal the concentration in whole blood. 

Before the experiment, the blood samples were incubated at +37 °C for 10 min. To obtain a control plasma sample, the red blood cells (RBC) were separated by centrifugation of whole blood (1500× *g*, +20 °C, 10 min). The nanoparticle suspension or a solution of doxorubicin in 1% of poloxamer 188 was added to fresh whole blood with anticoagulant and control plasma at concentrations of 10, 50, or 100 μg/mL and incubated with continuous shaking (+37 °C, 150 rpm). At predefined time points (5, 15, 30 min), the whole blood was centrifuged (1500× *g*, +20 °C, 10 min) and aliquots of b-plasma (from whole blood) were analyzed by HPLC as described above. The content of doxorubicin in the control plasma (*c_cp_*) was analyzed similarly. The RBC-to-plasma ratio (*K_RBC/Plasma_*) and blood-to-plasma ratio (*K_Blood/Plasma_*) were calculated according to Equations (5) and (6):

(5) $$K_{RBC/Plasma}=\frac{1}{H_{t}}\times \left(\frac{C_{cp}}{C_{p}}-1\right)+1$$

(6) $$K_{Blood/Plasma}=\frac{C_{cp}}{C_{p}},$$

where *C_cp_* represents the concentration of doxorubicin in the control plasma, *C_p_*—the concentration of doxorubicin in the b-plasma (from whole blood), *H_t_*—the hematocrit (average volume fraction of RBC in the whole blood equal to 0.4). The erythrocyte-bound doxorubicin fraction (%) after incubation in whole blood was calculated based on the obtained *K_Blood/Plasma_* values according to Equation (7):

(7) $$Erythrocyte-bound\;doxorubicin\;fraction\;\left(\%\right)=\left(1-\frac{1-H_{t}}{K_{Blood/Plasma}}\right)\times 100\%$$

All measurements were repeated three times.

### 3.9. Pharmacokinetic Study in Rats

#### 3.9.1. Animal Experiments

The in vivo experiments were performed following the European Convention for the Protection of Vertebrate Animals, Directives 86/609/EEC, recommendations of the FELASE working group (1986, 86/609/EEC, ISSN 03780 6978), and the National Standard of the Russian Federation R 53434-2009 ‘Good Laboratory Practice’.

All experiments were performed using adult female Wistar rats (150–250 g) obtained from the animal production unit of the Russian Academy of Sciences (Stolbovaya, Moscow Region, Russia). The rats were caged in groups of six and maintained on a standard 12-h light-dark cycle. They received standard laboratory food and water ad libitum throughout the study. The pharmacokinetic study was carried out using two different formulation prototypes compared to a solution of doxorubicin hydrochloride (control group) in water after intravenous administration to rats at a single dose of 5 mg of equivalent doxorubicin per kg of body weight. In the case of NanoCore-6.4, the pharmacokinetics was also studied for a dose of 2 mg/kg. The lyophilized particles (NanoCore-7.4 and NanoCore-6.4) were resuspended in an aqueous poloxamer 188 solution (1%) at a drug concentration of 2 mg/mL and were administered intravenously into the tail vein of the animals (*n* = 6 for each time point). 

At predefined time points (5, 15, 30 min, 1, 2, 4, 8, 24, and 48 h) the animals were euthanized with isoflurane and decapitated. The peripheral blood (a volume of 5–8 mL from each rat) was collected in K3-EDTA tubes and centrifuged immediately (1500× *g* for 10 min at +18 °C) to separate the plasma from the blood cells (Hettich^®^ Universal 320R centrifuge with swing-out rotor 1628, Andreas Hettich GmbH & Co. KG, Tuttlingen, Germany). One-half of each plasma sample was collected to separate free from the nanoparticle-bound fraction of doxorubicin by ultracentrifugation. For this, a volume of 1.5 mL of the plasma sample was filled into 10 mL centrifugation tubes and centrifuged for 30 min at 48,254× g (+4 °C, Avanti JXN-30). Afterward, a volume of 300 μL of the upper layer of the plasma sample was carefully transferred into 1.5 mL tubes. To preserve doxorubicin from degradation all plasma samples (total and free doxorubicin samples) were stored at −70 °C until further analysis. The stability of free and nanoparticle-bound doxorubicin was evaluated during a double freeze-thaw cycle in a pilot experiment (data not shown). 

#### 3.9.2. Sample Preparation Technique Used to Determine Free and Total Doxorubicin in Rat Plasma

The free and the total drug concentration were determined from rat plasma by HPLC using spectrofluorimetric detection. Before injection into the HPLC system, the proteins were precipitated using a mixture of a solution of 0.1% of formic acid in acetonitrile-DMSO (1:1). The concentrations were determined using a calibration curve for the reference doxorubicin substance in plasma according to the internal standard (daunorubicin). For a detailed description of the preparation of the calibration samples please refer to the Supplementary materials, Section S6. 

The plasma samples comprising free doxorubicin or total doxorubicin (including the free and the nanoparticle-bound fraction) were thawed at room temperature. A total volume of 220 μL was transferred into 1.5 mL tubes. A volume of 20 μL of the internal standard (daunorubicin) was added (2000 ng/mL) and mixed on a shaker at 1200 rpm for 2 min. Doxorubicin was extracted from the sample as described in the Supplementary materials, Section S6.

#### 3.9.3. Quantification of Doxorubicin

The assay was performed using an HPLC system (Agilent 1200) equipped with a diode array detector and a fluorescent detector and a ZORBAX Eclipse XDB-C18 column (80Å, 150 × 3 mm, 5.0 μm, Agilent Technologies, Santa Clara, CA, USA). The mobile phase consisted of a buffer (2.88 g/L SDS and 2.25 g/L orthophosphoric acid): acetonitrile (54:46) mixture delivered at a rate of 0.7 mL/min. The column temperature was +40 °C. For the assay, a sample of the organic phase (300 µL) obtained as described above was diluted with 300 µL of buffer solution. The injected volume was 5–60 μL. To calculate the concentrations the results obtained at λ_ex_ = 480 nm and λ_em_ = 550 nm were used. Additionally, detection at λ = 254 nm and λ_ex_/λ_em_ = 230/550 nm was used to identify the retention time of the peaks of doxorubicin and daunorubicin (IS). Plasma doxorubicin concentrations were calculated from two calibration curves using the chemical reference standard (CRS) of doxorubicin (EP CRS, 99.0%, EDQM). The first calibration curve was plotted in the concentration range of 4 to 400 ng/mL (S_Doxorubicin_/S_IS_ = 0.0047 × C_Doxorubicin_ + 0.007, C_Doxorubicin_ [ng/mL]). The second curve (S_Doxorubicin_/S_IS_ = 0.0043 × C_Doxorubicin_ + 0.4128, C_Doxorubicin_ [ng/mL]) was plotted in the concentration range of 400 to 20,000 ng/mL. The limit of quantitation (LOQ) was 0.007 µg/mL. In plasma, the coefficient of variation (CV) of precision was less than 20% and recovery was above 95%.

### 3.10. Analysis of Plasma Pharmacokinetics Using Non-Compartmental Modeling

Initially, all pharmacokinetic parameters were determined using Monolix Suite 2019R1 (Lixoft, Antony, France) using a non-compartmental modeling approach. This included the peak plasma concentration c_max_, the area under the plasma concentration versus time curve (AUC), the clearance (Cl) as well as the volume of distribution in the steady-state V.

### 3.11. Analyzing Plasma Pharmacokinetics Using the Physiologically-Based Nanocarrier Biopharmaceutics Model

The PBNB model combines conventional compartmental modeling with physiologically-based parameter ranges to estimate the free and the carrier-bound fraction of the drug [26]. Compared to previous investigations [26], the transit compartment, as well as the zero-order infusion, were replaced by bolus injection into the carrier compartment. This corresponds to the pharmacokinetic behavior expected for rodents. The analysis was carried out using Monolix Suite 2019R1 (Lixoft, Antony, France). A multi-compartment model was written in Mlxtran and executed using the Monolix software. In brief, the model is composed of three main compartments. The carrier compartment represents the carrier-bound fraction of the drug which, due to reduced mobility compared to the free compound, exhibits a smaller volume of distribution (V_DC_). V_DC_ was assumed to correspond to the blood plasma volume of rats [26,48]. The accumulation of the carrier occurring due to the interactions of the delivery system with cells or tissues is represented by a first-order elimination process from the carrier compartment. This elimination process is reported in the carrier half-life (t_½_) and is widely responsible for the accumulated fraction of the drug. A second pathway leads from this carrier compartment into the compartment representing the released fraction of the drug. A release process following the 3RPT model was assumed [26]. Initially, the reciprocal powered time model was developed for quality control purposes and enables a wide range of release behaviors to be accurately described. Although it is based on a dissolution and diffusion process, the model introduces two variables (m, b) to account for the influence of hydrodynamics and aggregation of particles during the release process [36]. The 3RPT model introduces a third release parameter (c) to model biorelevant release in absence of sink conditions [50].

The free drug is accurately described by a conventional two-compartment model following first-order kinetics and comprising the central compartment as well as one periphery compartment (k_12_, k_21_). Further, a first-order elimination process is assumed (k_F_). An illustration of this model is presented in Figure 6. All parameters were limited to predefined ranges as presented in Table 7. 

Based on previous findings, the half-life of the carrier was set to a maximum of 1 h. The distribution and elimination parameters (k_F_, k_12_, k_21_), as well as the volume of distribution of the free drug (V_DF_), were set to the pharmacokinetic parameters observed for the control group treated with an aqueous solution of doxorubicin. A traditional two-compartment model following a bolus injection and linear elimination kinetics calculated using the PKanalix software (Lixoft, Antony, France). Additionally, the in vivo drug release and the targeting capability were calculated as described previously [26]. In brief, the cumulative in vivo drug release (%) is defined as the dose fraction of the drug being released from the carrier while the targeting capability (*F_target_*, %) represents the dose fraction that is accumulated before it was being released from the carrier (Equation (8)).

(8) $$F_{target}\left(\%\right)=\frac{m_{accum}}{Dose}\times 100\%$$

### 3.12. Data Analysis and Statistics

Statistical analysis of the data was performed with Statsoft Statistica 8.1 (StatSoft Inc., Tulsa, OK, USA) software using the Student t-test; statistical significance level was set at *p* < 0.05. The characterization of particle size, size distribution, and zeta potential was carried out in quadruplicates while all in vitro measurements were conducted in triplicates. For the animal experiments, all mice were caged and treated in groups of six.

## 4. Conclusions

Characterization of the drug release in combination with a detailed analysis of the pharmacokinetic features of the formulation prototypes NanoCore-6.4 and NanoCore-7.4 shed new light on the intertwined processes underlying targeted delivery of doxorubicin. Lipid-like nanoparticles released doxorubicin at different rates in vitro and in vivo highlighting the effectiveness of the design strategy. Although our findings indicate a difference in the drug release leading to bioequivalent formulations, the expectations of a quality-by-design approach were fulfilled. A shift in one critical process parameter, the pH during synthesis, led to an improved target capability in vivo. Keeping in mind that release assays may tend to overestimate the differences between two formulations, further studies will now focus on formulation development based on the validated in vitro assay.

## Figures and Tables

**Table 1:** Physicochemical properties of the NanoCore-7.4 and NanoCore-6.4 after freeze-drying (*n* = 3, mean ± standard deviation).

Formulation	pH of External Aqueous Phase	Encapsulation Efficiency (%)	Drug Load (%)	Size Distribution				Zeta Potential (mV)
				Mean Particle Diameter (nm)	PDI	Intensity (nm)	Volume (nm)	
NanoCore-7.4	7.4	91 ± 1	9.1 ± 0.3	105 ± 12	0.144 ± 0.011	118 (100%)	94 (100%)	−10.46 ± 1.33
NanoCore-6.4	6.4	80 ± 1	7.8 ± 0.1	137 ± 7	0.301 ± 0.069	157 (94%)	119 (91%)	−6.40 ± 2.27

**Table 2:** Physicochemical properties of the NanoCore-7.4 and NanoCore-6.4 before freeze-drying (*n* = 3, mean ± standard deviation).

Formulation	pH of External Aqueous Phase	Size Distribution			
		Mean Particle Diameter (nm)	PDI	Intensity (nm)	Volume (nm)
NanoCore-7.4	7.4	93 ± 7	0.092 ± 0.012	106 (100%)	85 (100%)
NanoCore-6.4	6.4	89 ± 12	0.110 ± 0.003	99.8 (100%)	80 (100%)

**Table 3:** Mathematical description and comparison of the 48 h release profiles of doxorubicin from NanoCore-7.4 and NanoCore-6.4 nanoparticles using Korsmeyer-Peppas model.

Formulation	Time, h	K	n	R^2^
NanoCore-7.4	1–6	0.1792	0.1745	0.9199
	1–48	0.1629	0.3152	0.9030
	1–120	0.1581	0.3115	0.9584
NanoCore-6.4	1–6	0.3614	0.0521	0.8603
	1–48	0.3424	0.1865	0.8726
	1–120	0.3009	0.2459	0.9293

**Table 4:** Average values of erythrocyte-bound doxorubicin fraction (%).

Time	Doxorubicin Concentration (μg/mL)								
	10			50			100		
	Free Doxorubicin	Nano- Core-7.4	Nano- Core-6.4	Free Doxorubicin	Nano- Core-7.4	Nano- Core-6.4	Free Doxorubicin	Nano- Core-7.4	Nano- Core-6.4
**5 min**	35	33	36	33	32	33	33	31	32
**15 min**	59	49	57	62	46	58	63	49	58
**30 min**	60	50	55	61	47	50	64	47	57

**Table 5:** Pharmacokinetic parameters identified by the PBNB model using a more restrictive range for the carrier half-life.

Formulation	Parameter	Mean	Min	Max	SD	CV
NanoCore-7.4_total_ (5 mg/kg)	Half-life (h)	0.374	0.375	0.372	0.001	0.003
	m	0.468	0.468	0.466	0.000	0.001
	b	0.832	0.840	0.826	0.005	0.006
	c	9.052	9.058	9.046	0.004	0.000
	V_DC_ (mL)	11.935	12.018	11.862	0.050	0.004
	F_target_ (%)	7.415	7.615	7.310	0.110	0.015
NanoCore-6.4_total_ (5 mg/kg)	Half-life (h)	0.395	0.401	0.391	0.004	0.009
	m	0.460	0.470	0.449	0.008	0.017
	b	0.880	0.883	0.877	0.002	0.002
	c	11.227	11.256	11.173	0.031	0.003
	V_DC_ (mL)	10.521	10.595	10.433	0.051	0.005
	F_target_ (%)	5.899	6.057	5.740	0.106	0.018
NanoCore-6.4_total_ (2 mg/kg)	Half-life (h)	0.388	0.392	0.384	0.002	0.006
	m	0.413	0.430	0.398	0.012	0.029
	b	0.870	0.874	0.866	0.002	0.003
	c	11.364	11.405	11.301	0.035	0.003
	V_DC_ (mL)	10.795	10.871	10.723	0.045	0.004
	F_target_ (%)	5.143	5.426	4.885	0.183	0.036

**Table 6:** Summary of the different release models applied to analyze the in vitro release data. In this summary, *M_t_* represents the amount of the drug released over a certain time (*t*) and *M_∞_* represents the total drug amount. The parameter n denotes the diffusion coefficient in the Korsmeyer-Peppas equation. In the 3RPT model, *M_t_*/*M*_∞_ represents the fraction of the dose released, *m*—the time at which 50% of the plateau phase has been reached, parameter *b* determines the shape of the profile, *c*—the released fraction in the plateau phase.

Zero-order model	$\frac{M_{t}}{M_{\infty }}=K_{0}\times t$	Higuchi model	$\frac{M_{t}}{M_{\infty }}=K_{H}\times t^{\frac{1}{2}}$
First-order model	$\ln\left(1-\frac{M_{t}}{M_{\infty }}\right)=-K_{1}\times t$	Hickson-Crowell model	${\left(1-\frac{M_{t}}{M_{\infty }}\right)}^{\frac{1}{3}}=1-K_{\beta }\times t$
Korsmeyer-Peppas model	$\frac{M_{t}}{M_{\infty }}=K_{KP}\times t^{n}$	3-parametric reciprocal powered time model	$\frac{M_{t}}{M_{\infty }}=\frac{t^{b}}{t^{b}+m}\times c$

**Table 7:** Initial estimates used by the PBNB model for data analysis. Two simulations were carried with different initial estimates for the half-life (a and b). The second half-life as well as the pharmacokinetic parameters of the free drug were obtained by pharmacokinetic analysis and not extracted from a literature source.

Parameter	Range
V_DC_ (L) [48]	8.325 ± 4.03 (ω: 0.5)
t_½_ (h) ^a^ [46,53]	0.5 ± 0.71 (ω: 0.5)
t_½_ (h) ^b^	0.364 ± 0.017 (ω: 0.5)
k_F_ (h^−1^)	1.074 ± 0.46 (ω: 0.5)
k_12_ (h^−1^)	13.77 ± 2.62 (ω: 0.5)
k_21_ (h^−1^)	0.68 ± 0.21 (ω: 0.5)
V_DF_ (mL)	787.69 ± 293.13 (ω: 0.5)

## Supplementary Materials

The following are available online at, S1: Optimization of the method for evaluation of the release profile of doxorubicin in model media in vitro; S2: Mathematical description and comparison of the release profiles of doxorubicin from nanoparticles; S3: Quantification of doxorubicin in human plasma; S4: Optimization of the method for evaluation of the kinetics of doxorubicin release in plasma; S5: Quantification of the blood-to-plasma rate ratio (*K_Blood/Plasma_*) and the erythrocyte-to-plasma rate ratio (*K_RBC/Plasma_*); S6: Quantification of doxorubicin in rat plasma; S7: Non-compartmental analysis of pharmacokinetics.

## References

[1] Hare J.I., Lammers T., Ashford M.B., Puri S., Storm G., Barry S.T. (2017). Challenges and strategies in anti-cancer nanomedicine development: An industry perspective. Adv. Drug Deliv. Rev..

[2] Siddique S., Chow J.C.L. (2020). Application of Nanomaterials in Biomedical Imaging and Cancer Therapy. Nanomaterials.

[3] Wacker M. (2013). Nanocarriers for intravenous injection--the long hard road to the market. Int. J. Pharm..

[4] Wacker M.G. (2019). Frontiers in pharmaceutical nanotechnology. Beilstein. J. Nanotechnol..

[5] Cheo S.T.T., Lim G.H., Lim K.H.C. (2017). Glioblastoma multiforme outcomes of 107 patients treated in two Singapore institutions. Singap. Med. J..

[6] Feczko T., Piiper A., Ansar S., Blixt F.W., Ashtikar M., Schiffmann S., Ulshofer T., Parnham M.J., Harel Y., Israel L.L. (2019). Stimulating brain recovery after stroke using theranostic albumin nanocarriers loaded with nerve growth factor in combination therapy. J. Control. Release.

[7] Salvalaio M., Rigon L., Belletti D., D’Avanzo F., Pederzoli F., Ruozi B., Marin O., Vandelli M.A., Forni F., Scarpa M. (2016). Targeted Polymeric Nanoparticles for Brain Delivery of High Molecular Weight Molecules in Lysosomal Storage Disorders. PLoS ONE.

[8] Petri B., Bootz A., Khalansky A., Hekmatara T., Muller R., Uhl R., Kreuter J., Gelperina S. (2007). Chemotherapy of brain tumour using doxorubicin bound to surfactant-coated poly(butyl cyanoacrylate) nanoparticles: Revisiting the role of surfactants. J. Control. Release.

[9] Wohlfart S., Khalansky A.S., Gelperina S., Maksimenko O., Bernreuther C., Glatzel M., Kreuter J. (2011). Efficient chemotherapy of rat glioblastoma using doxorubicin-loaded PLGA nanoparticles with different stabilizers. PLoS ONE.

[10] Ganipineni L.P., Danhier F., Preat V. (2018). Drug delivery challenges and future of chemotherapeutic nanomedicine for glioblastoma treatment. J. Control Release.

[11] Kim S.S., Harford J.B., Pirollo K.F., Chang E.H. (2015). Effective treatment of glioblastoma requires crossing the blood-brain barrier and targeting tumors including cancer stem cells: The promise of nanomedicine. Biochem. Biophys. Res. Commun..

[12] Zhao M., van Straten D., Broekman M.L.D., Preat V., Schiffelers R.M. (2020). Nanocarrier-based drug combination therapy for glioblastoma. Theranostics.

[13] Kreuter J., Ramge P., Petrov V., Hamm S., Gelperina S.E., Engelhardt B., Alyautdin R., von Briesen H., Begley D.J. (2003). Direct evidence that polysorbate-80-coated poly(butylcyanoacrylate) nanoparticles deliver drugs to the CNS via specific mechanisms requiring prior binding of drug to the nanoparticles. Pharm. Res..

[14] Kreuter J., Shamenkov D., Petrov V., Ramge P., Cychutek K., Koch-Brandt C., Alyautdin R. (2002). Apolipoprotein-mediated transport of nanoparticle-bound drugs across the blood-brain barrier. J. Drug Target.

[15] Steiniger S.C., Kreuter J., Khalansky A.S., Skidan I.N., Bobruskin A.I., Smirnova Z.S., Severin S.E., Uhl R., Kock M., Geiger K.D. (2004). Chemotherapy of glioblastoma in rats using doxorubicin-loaded nanoparticles. Int. J. Cancer.

[16] Zensi A., Begley D., Pontikis C., Legros C., Mihoreanu L., Wagner S., Buchel C., von Briesen H., Kreuter J. (2009). Albumin nanoparticles targeted with Apo E enter the CNS by transcytosis and are delivered to neurones. J. Control. Release.

[17] Zensi A., Begley D., Pontikis C., Legros C., Mihoreanu L., Buchel C., Kreuter J. (2010). Human serum albumin nanoparticles modified with apolipoprotein A-I cross the blood-brain barrier and enter the rodent brain. J. Drug Target.

[18] Pereverzeva E., Treschalin I., Bodyagin D., Maksimenko O., Kreuter J., Gelperina S. (2008). Intravenous tolerance of a nanoparticle-based formulation of doxorubicin in healthy rats. Toxicol. Lett..

[19] Sulheim E., Iversen T.-G., To Nakstad V., Klinkenberg G., Sletta H., Schmid R., Hatletveit A.R., Wågbø A.M., Sundan A., Skotland T. (2017). Cytotoxicity of Poly(Alkyl Cyanoacrylate) Nanoparticles. Int. J. Mol. Sci..

[20] Khalin I., Severi C., Heimburger D., Wehn A., Hellal F., Reisch A., Klymchenko A.S., Plesnila N. (2020). Highly fluorescent biodegradable PLGA nano-carriers allow real-time tracking of individual particles in vivo. bioRxiv.

[21] Wohlfart S., Khalansky A.S., Gelperina S., Begley D., Kreuter J. (2011). Kinetics of transport of doxorubicin bound to nanoparticles across the blood-brain barrier. J. Control. Release.

[22] Gelperina S., Maksimenko O., Khalansky A., Vanchugova L., Shipulo E., Abbasova K., Berdiev R., Wohlfart S., Chepurnova N., Kreuter J. (2010). Drug delivery to the brain using surfactant-coated poly(lactide-co-glycolide) nanoparticles: Influence of the formulation parameters. Eur. J. Pharm. Biopharm..

[23] Filon O., Krivorotko P., Kobyakov G., Razjivina V., Maximenko O., Gelperina S., Kreuter J. (2017). A phase I study of safety and pharmacokinetics of NanoBB-1-Dox in patients with advanced solid tumors. J. Clin. Oncol..

[24] Rodrigues de Azevedo C., von Stosch M., Costa M.S., Ramos A.M., Cardoso M.M., Danhier F., Préat V., Oliveira R. (2017). Modeling of the burst release from PLGA micro- and nanoparticles as function of physicochemical parameters and formulation characteristics. Int. J. Pharm..

[25] Janas C., Mast M.P., Kirsamer L., Angioni C., Gao F., Mantele W., Dressman J., Wacker M.G. (2017). The dispersion releaser technology is an effective method for testing drug release from nanosized drug carriers. Eur. J. Pharm. Biopharm..

[26] Nagpal S., Braner S., Modh H., Tan A.X.X., Mast M.P., Chichakly K., Albrecht V., Wacker M.G. (2020). A physiologically-based nanocarrier biopharmaceutics model to reverse-engineer the in vivo drug release. Eur. J. Pharm. Biopharm..

[27] Fulop Z., Gref R., Loftsson T. (2013). A permeation method for detection of self-aggregation of doxorubicin in aqueous environment. Int. J. Pharm..

[28] Maksimenko O., Malinovskaya J., Shipulo E., Osipova N., Razzhivina V., Arantseva D., Yarovaya O., Mostovaya U., Khalansky A., Fedoseeva V. (2019). Doxorubicin-loaded PLGA nanoparticles for the chemotherapy of glioblastoma: Towards the pharmaceutical development. Int. J. Pharm..

[29] Kreuter J. (2014). Drug delivery to the central nervous system by polymeric nanoparticles: What do we know?. Adv. Drug Deliv. Rev..

[30] Pereverzeva E., Treschalin I., Treschalin M., Arantseva D., Ermolenko Y., Kumskova N., Maksimenko O., Balabanyan V., Kreuter J., Gelperina S. (2019). Toxicological study of doxorubicin-loaded PLGA nanoparticles for the treatment of glioblastoma. Int. J. Pharm..

[31] Fugit K.D., Xiang T.X., Choi du H., Kangarlou S., Csuhai E., Bummer P.M., Anderson B.D. (2015). Mechanistic model and analysis of doxorubicin release from liposomal formulations. J. Control. Release.

[32] Nothnagel L., Wacker M.G. (2018). How to measure release from nanosized carriers?. Eur. J. Pharm. Sci..

[33] Feczko T., Piiper A., Pleli T., Schmithals C., Denk D., Hehlgans S., Rodel F., Vogl T.J., Wacker M.G. (2019). Theranostic Sorafenib-Loaded Polymeric Nanocarriers Manufactured by Enhanced Gadolinium Conjugation Techniques. Pharmaceutics.

[34] Hines D.J., Kaplan D.L. (2013). Poly(lactic-co-glycolic) acid-controlled-release systems: Experimental and modeling insights. Crit. Rev. Ther. Drug Carr. Syst..

[35] Ritger P.L., Peppas N.A. (1987). A simple equation for description of solute release II. Fickian and anomalous release from swellable devices. J. Control. Release.

[36] Mohammadi G., Barzegar-Jalali M., Valizadeh H., Nazemiyeh H., Barzegar-Jalali A., Siahi Shadbad M.R., Adibkia K., Zare M. (2010). Reciprocal powered time model for release kinetic analysis of ibuprofen solid dispersions in oleaster powder, microcrystalline cellulose and crospovidone. J. Pharm. Pharm. Sci..

[37] Wallenwein C.M., Nova M.V., Janas C., Jablonka L., Gao G.F., Thurn M., Albrecht V., Wiehe A., Wacker M.G. (2019). A dialysis-based in vitro drug release assay to study dynamics of the drug-protein transfer of temoporfin liposomes. Eur. J. Pharm. Biopharm..

[38] Laubrock N., Hempel G., Schulze-Westhoff P., Würthwein G., Flege S., Boos J. (2000). The stability of doxorubicin and ldarubicin in plasma and whole blood. Chromatographia.

[39] Piazza E., Broggini M., Trabattoni A., Natale N., Libretti A., Donelli M.G. (1981). Adriamycin distribution in plasma and blood cells of cancer patients with altered hematocrit. Eur. J. Cancer Clin. Oncol..

[40] Anselmo A.C., Gupta V., Zern B.J., Pan D., Zakrewsky M., Muzykantov V., Mitragotri S. (2013). Delivering nanoparticles to lungs while avoiding liver and spleen through adsorption on red blood cells. ACS Nano.

[41] Colombo T., Broggini M., Garattini S., Donelli M.G. (1981). Differential adriamycin distribution to blood components. Eur. J. Drug Metab. Pharm..

[42] Yu S., Li S., Yang H., Lee F., Wu J.T., Qian M.G. (2005). A novel liquid chromatography/tandem mass spectrometry based depletion method for measuring red blood cell partitioning of pharmaceutical compounds in drug discovery. Rapid Commun. Mass Spectrom..

[43] Kalamaridis D., DiLoreto K., Caldwell G.W., Yan Z. (2014). Drug Partition in Red Blood Cells. Optimization in Drug Discovery: In Vitro Methods.

[44] Rahman A., Carmichael D., Harris M., Roh J.K. (1986). Comparative pharmacokinetics of free doxorubicin and doxorubicin entrapped in cardiolipin liposomes. Cancer Res..

[45] Le Ray A.M., Vert M., Gautier J.C., Benoît J.P. (1994). Fate of [14C]poly(DL-lactide-co-glycolide) nanoparticles after intravenous and oral administration to mice. Int. J. Pharm..

[46] Rafiei P., Haddadi A. (2017). Docetaxel-loaded PLGA and PLGA-PEG nanoparticles for intravenous application: Pharmacokinetics and biodistribution profile. Int. J. Nanomed..

[47] Ishihara T., Maeda T., Sakamoto H., Takasaki N., Shigyo M., Ishida T., Kiwada H., Mizushima Y., Mizushima T. (2010). Evasion of the accelerated blood clearance phenomenon by coating of nanoparticles with various hydrophilic polymers. Biomacromolecules.

[48] Lee H.B., Blaufox M.D. (1985). Blood volume in the rat. J. Nucl. Med..

[49] Yuan D., He H., Wu Y., Fan J., Cao Y. (2019). Physiologically Based Pharmacokinetic Modeling of Nanoparticles. J. Pharm. Sci..

[50] Nothnagel L., Jung F., Rossmanith T., Thurn M., Ashtikar M., Geisslinger G., Parnham M.J., Wacker M.G. (2019). Predictive PBPK modeling as a tool in the formulation of the drug candidate TMP-001. Eur. J. Pharm. Biopharm..

[51] Malinovskaya Y., Melnikov P., Baklaushev V., Gabashvili A., Osipova N., Mantrov S., Ermolenko Y., Maksimenko O., Gorshkova M., Balabanyan V. (2017). Delivery of doxorubicin-loaded PLGA nanoparticles into U87 human glioblastoma cells. Int. J. Pharm..

[52] Heron M.W., Paton B.C. (1968). A method for measuring a nonionic surface-active agent (Pluronic F-68) in biological fluids. Anal. Biochem..

[53] Bian X., Liang S., John J., Hsiao C.-H., Wei X., Liang D., Xie H. (2013). Development of PLGA-based itraconazole injectable nanospheres for sustained release. Int. J. Nanomed..

