# Supplementary material for: Exploring the Interplay between Drug Release and Targeting of Lipid-Like Polymer Nanoparticles Loaded with Doxorubicin
Source: Molecules. 2021 Feb 5;26(4):831. doi: 10.3390/molecules26040831 (PMC7915178; doi:10.3390/molecules26040831)
Supplement: Supplementary file 1 [file molecules-26-00831-s001.pdf]

Supplementary materials to the article:

Exploring the interplay between drug release and targeting of lipid-like polymer nanoparticles loaded with doxorubicin

Tatyana Kovshova, Nadezhda Osipova, Anna Alekseeva, Julia Malinovskaya, Alexey Belov, Andrey Budko, Galina Pavlova, Olga Maksimenko, Shakti Nagpal, Svenja Braner, Harshvardhan Modh, Vadim Balabanyan, Matthias G. Wacker and Svetlana Gelperina

S1. Optimization of the method for evaluation of the release profile of doxorubicin in model media *in vitro*

The *in vitro* release is a key characteristic of nanocarriers relevant for the *in vivo* efficacy of the formulation. To quantify the release rate a broad array of methods and media were evaluated.

During our initial studies utilizing the 'dialysis bag' method, significant retention of doxorubicin was responsible for the limited sensitivity of the assay. Although the membrane was preincubated with doxorubicin solution before the experiment (data not shown), there was a considerable lag time before the drug was detected in the acceptor compartment.

Therefore, further experiments were performed using centrifugation to separate the free drug from the nanoparticle-bounded fraction.

To avoid the influence of mechanical shear forces on the outcome of our investigation, different centrifugation rates (15,000 × g and 48,154 × g) were compared. Also, to assess the effect of the degree of dilution of the nanosuspensions on the release rate of doxorubicin, an *in vitro* profile was studied for dilutions of 1:5 (to the concentration of doxorubicin of approximately 340 μg/ml) and 1:25 (to the concentration of doxorubicin of approximately 68 μg/ml). Both formulations were tested over 120 h.

The shear forces applied during the separation as well as the dilution factor of the drug delivery system had a strong impact on the release profile. The amount of doxorubicin detected in the supernatants of NanoCore-7.4 appeared to be higher at lower centrifugation rates as compared to high centrifugation rates, suggesting an incomplete separation of the particles from the dissolved drug (Figure 1). Indeed, the quantification of PLGA using capillary electrophoresis, confirmed a considerable difference in the polymer content between the supernatants obtained at lower and higher centrifugation rates. In the case of NanoCore-7.4, 26% (15,000 × g) and 10% (48,254 × g) of the initial polymer content (taking into account 3-5% of a soluble monomer fraction of PLGA in the nanoparticle samples) were found. This considerable difference mandates the use of a higher centrifugation rate for nanoparticle separation. With regards to the dilution of the nanosuspension, the influence of the centrifugation method was even more important at a lower (1:5) dilution as compared to a higher dilution (1:25). Thus, in the case of NanoCore-7.4 diluted 5-fold, the concentrations of doxorubicin in the supernatants obtained after centrifugation at 15,000 × g and 48,254 × g differed by 12%, while at 1:25 dilution the difference was only 6% (Figure 1, 1 h of incubation). The lower difference observed at a higher dilution suggests a strong 'burst effect'. Similar results were obtained for NanoCore-6.4 (data not shown). The difference between the two formulations was more pronounced at a higher dilution (1:25) (data not shown).

A reliable method was developed for assessing the release of doxorubicin from PLGA nanoparticles into a model media. The optimal conditions included a 1% poloxamer 188 aqueous solution as the release medium, the dilution of the suspension 1:25 (to the concentration of doxorubicin of approximately 68 μg/ml) and ultracentrifugation (acceleration of centrifugation – 48,254 × g) as the most suitable technique for the nanoparticle separation.

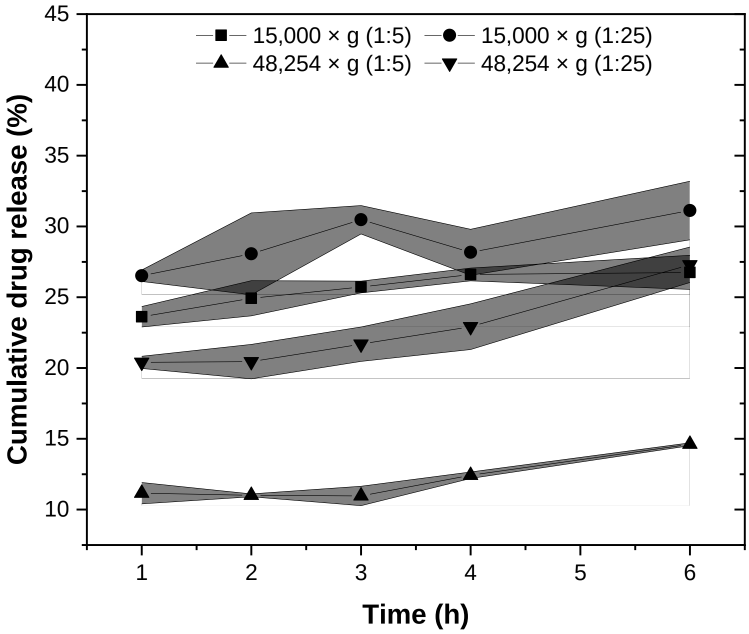

Figure 1: Release kinetics determined over 6 h for NanoCore-7.4 in 1% poloxamer 188 using 15,000 x g with dilutions of 1:5 (■) and 1:25 (●) as well as at 48,254 x g with dilutions of 1:5 (▲) and 1:25 (▼), n = 3.

S2. Mathematical description and comparison of the release profiles of doxorubicin from nanoparticles

Average kinetic parameters of the 120 h *in vitro* release of doxorubicin from nanoformulations in 1% poloxamer 188 and separately for the «burst-effect» phase (1-6 h) and the subsequent slow release phase (6-120 h) calculated using the first-order, Higuchi and Korsmeyer-Peppas models were described in Table 1. The mathematical description and comparison of the 48-h release profiles of doxorubicin from NanoCore-7.4 and NanoCore-6.4 nanoparticles using the zero and the first-order models, as well as the Higuchi, Hickson-Crowell, the Korsmeyer-Peppas models are described in Table 2.

Table 1: Mathematical description of doxorubicin 120-h release from NanoCore-7.4 and NanoCore-6.4 in a 1% poloxamer 188 solution (ultracentrifugation, n=3)

NP type	Time, h	Korsmeyer-Peppas model			Higuchi model		First-order model	
		K	n	R^2	K	R^2	K	R^2
NanoCore-7.4 (n = 3)	1-6	0.1792 ± 0.0367	0.1745 ± 0.0320	0.9199 ± 0.0361	0.0463 ± 0.0041	0.9504 ± 0.0148	-0.0172 ± 0.0024	0.9599 ± 0.0323
	6-120	0.1264 ± 0.0255	0.3740 ± 0.0312	0.9698 ± 0.0109	0.0586 ± 0.0089	0.9536 ± 0.0355	-0.0095 ± 0.0030	0.9348 ± 0.0672
	1-120	0.1581 ± 0.0311	0.3115 ± 0.0285	0.9584 ± 0.002	0.0629 ± 0.0129	0.9576 ± 0.0311	-0.0112 ± 0.0019	0.9713 ± 0.0395
NanoCore-6.4 (n = 3)	1-6	0.3614 ± 0.0057	0.0521 ± 0.0078	0.8603 ± 0.0229	0.0251 ± 0.0035	0.9055 ± 0.0017	-0.0113 ± 0.0008	0.9697 ± 0.0392
	6-120	0.2360 ± 0.0243	0.3167 ± 0.0143	0.9671 ± 0.0543	0.0683 ± 0.0054	0.9083 ± 0.0134	-0.0336 ± 0.0021	0.9995 ± 0.0143
	1-120	0.3009 ± 0.0214	0.2459 ± 0.0134	0.9293 ± 0.0321	0.0717 ± 0.0021	0.9539 ± 0.0365	-0.0332 ± 0.0043	0.9991 ± 0.0543

Table 2: Mathematical description and comparison of the 48-h release profiles of doxorubicin from NanoCore-7.4 and NanoCore-6.4 nanoparticles.

NP type	Time, h	Zero-order model		First order model		Higuchi model		Hickson-Crowell		Korsmeyer-Peppas model		
		K	R^2	K	R^2	K	R^2	K	R^2	K	n	R^2
NanoCore-7.4	1-48	0.0096	**0.9953**	-0.0173	0.9906	0.0763	0.9699	-0.0047	0.9944	0.1629	**0.3152**	0.9030
NanoCore-6.4	1-48	0.0089	**0.9930**	-0.0243	0.9643	0.0699	0.9509	-0.0057	0.9772	0.3424	**0.1865**	0.8726

S3. Quantification of doxorubicin in human plasma

1. Preparation of stock solutions, calibration, and doxorubicin-loaded nanoparticle samples

Stock solutions of doxorubicin hydrochloride (1 mg/mL, EP CRS, 99.0%, EDQM) and the internal standard (IS) daunorubicin (10 mg/mL, EP CRS, 98.5%, EDQM) were prepared by dissolving the weighted amounts of the drugs in water. The analyte stock solutions were divided into 5 aliquots and placed at <-70 ° C for storage. A volume of 100 µL of the IS stock solution was diluted with 25 mL of a 0.1% aqueous solution of formic acid to obtain the IS working solution (40 µg/mL), which was then divided into 20 aliquots and stored at <-70 °C.

The calibration standards were prepared using pooled human plasma with doxorubicin concentrations ranging from 2 to 100 µg/mL. The preparation procedure of calibration stock solutions and 10X calibration standard solutions of doxorubicin is illustrated in Table 3.

Table 3: Preparation of 10X calibration solutions

Preparation of calibration stock solutions:					
Doxorubicin concentration, µg/mL		**Preparation**			
100		40 µL doxorubicin stock solution [1 mg/mL] + 360 µL [ACN:H_2O (1:1) + 0.1% formic acid]			
Preparation of 10X calibration standard solutions					
Calibration standard	Doxorubicin concentration in plasma, µg/mL	Doxorubicin concentration in 10X solution, µg/mL	Doxorubicin concentration in stock solution, µg/mL	Volume of stock solution, µL	Volume of ACN:H_2O (1:1) + 0.1% formic acid solution, µL
K1	2	20	100	20	80
K2	4	40	100	40	60
K3	10	100	100	100	-
K4	20	200	1,000	20	120
K5	40	400	1,000	40	60
K6	80	800	1,000	80	20
K7	100	1,000	1,000	100	-

Stock suspensions of doxorubicin-loaded nanoparticles (1 mg/mL) were obtained by resuspending the freeze-dried samples in a 1% aqueous solution of poloxamer 188 followed by 30 min of incubation at ambient temperature. The preparation procedure of 10X solutions of doxorubicin-loaded nanoparticles is illustrated in Table 4.

Table 4: Preparation of 10X nanoparticle-bound doxorubicin suspensions

Doxorubicin concentration in plasma, µg / mL	Doxorubicin concentration in 10X suspensions, µg/mL	Doxorubicin concentration in stock suspensions, µg/mL	Volume of stock suspensions, µL	Volume of ACN:H_2O (1:1) + 0.1% formic acid solution, µL
10	100	1,000	100	900
50	500	1,000	500	500
100	1,000	1,000	500	-

2. Sample preparation technique used to determine total doxorubicin concentration in human plasma for calibration and PLGA nanoparticles samples

Aliquots with a volume of 20 µL of 10X calibration standard solutions of doxorubicin or 10X doxorubicin-loaded nanoparticle suspensions were added to 200 µl of plasma in 1.5 mL tubes and gently mixed for 2 min. A volume of 80 µL of the IS working solution (daunorubicin, 40 µg/mL) was added to the samples except for the blank samples (samples without analytes). In the case of blank samples, a volume of 20 µL of blank solvent (0.1% of formic acid in acetonitrile-water (1:1)) was added. For extraction of doxorubicin (and IS), 300 µL of a cooled (-20 °C) 0.1% solution of formic acid in acetonitrile was added, followed by shaking of the samples at 1,200 rpm for 2 min. Afterwards, a volume of 300 µL of DMSO was

added. After shaking the samples at 1,200 rpm for 8 min, they were centrifuged for 30 min at 4,000 rpm and a temperature of +4°C in an Eppendorf 5804R centrifuge (Eppendorf AG, Hamburg, Germany). A volume of 250 µL of the supernatant was mixed with 250 µL of a solution of 62,5% aqueous acetonitrile supplemented with 0.1% formic acid and analyzed by HPLC (Shimadzu System, Symmetry C18 column, 5 µm, 3.9 x 150 mm) using spectrophotometric detection (at λ_{ex}=254 nm). Concentrations of total doxorubicin in the plasma were calculated using a calibration curve with normalization to the internal standard (daunorubicin).

For comparison, extraction of doxorubicin was carried out using acetonitrile supplemented with 0.1% of formic acid (extraction without DMSO). For extraction of doxorubicin (and IS) a volume of 600 µL of the cooled (-20 °C) solution was added to the samples. After shaking the samples at 1,200 rpm for 10 min, they were centrifuged for 30 min at 4,000 rpm and a temperature of +4 °C in an Eppendorf 5804R centrifuge (Eppendorf AG, Hamburg, Germany). A volume of 250 µL of the supernatant was mixed with 250 µL of a solution of 25% acetonitrile supplemented with 0.1% formic acid before further analysis by HPLC.

3. Sample preparation technique used to determine free doxorubicin concentration in human plasma

To study the effect of the centrifugal acceleration (15,000 x g or 48,254 x g) on the percentage of free doxorubicin in the blood plasma, the pooled plasma was thawed at room temperature and vortexed. For the samples centrifuged at 15,000 x g, a volume of 600 µL of plasma was transferred into 1.5 mL microtubes supplemented with 60 µL of a 10X nanoparticle doxorubicin suspension and was gently mixed. Samples were centrifuged for 30 min at the predefined acceleration and a temperature of +4°C in an Eppendorf 5804R centrifuge (Eppendorf AG, Hamburg, Germany). For samples centrifuged at 48,254 x g, a volume of 1 mL of the pooled plasma was transferred to 10 mL centrifuge tubes, a volume of 100 µL was added to 10X nanoparticle suspension and gently mixed. Samples were centrifuged for 30 min at the predefined acceleration and a temperature of +4°C in an Avanti JXN-30 centrifuge (Beckman Coulter, Pasadena, USA).

For both preparations, a volume of 220 µL of the supernatants was carefully transferred into 1.5 mL tubes, and the sample preparation was performed as described previously for total doxorubicin (using acetonitrile supplemented with 0.1% formic acid or acetonitrile: DMSO (1:1) supplemented with 0.1% formic acid).

S4. Optimization of the method for evaluation of the kinetics of doxorubicin release in plasma

The release of doxorubicin from the NanoCore-7.4 and NanoCore-6.4 nanoparticles in human plasma was evaluated by determination of the free and the total doxorubicin concentration using an HPLC method. During optimization, were used various extractants, as well as different modes of centrifugation for determination of the free and the total doxorubicin concentration in plasma. The data on the development of a sample preparation method for the determination of both fractions of doxorubicin by HPLC are given below.

As shown before, acetonitrile alone does not completely dissolve the nanoparticles, while the addition of DMSO considerably increases the efficiency of doxorubicin extraction (from 83-93% to 97-102% depending on the concentration of nanoparticles). Also, for both extractants, the extraction was decreasing with an increase in the nanoparticle concentration. In the present approach, doxorubicin (free and nanoparticle-bounded) was extracted at three different concentrations from human plasma using a mixture of acetonitrile and DMSO (1:1) supplemented with 0.1% of formic acid. The percentage of extracted total doxorubicin varied between 97.16 ± 2.89% and 101.90 ± 0.30% for 100 µg/mL and 10 µg/mL, respectively.

To enable a quantification of the doxorubicin concentration from the blood plasma, a freezing procedure was developed to keep the drug stable until further analysis. To assess the stability of the nanoparticulate formulations during freezing-thawing a 10-fold concentrated NanoCore-7.4 nanoparticle suspension (500 µg/mL) was added to plasma resulting in a nominal concentration of 50 µg/mL of doxorubicin. After freezing this sample for 3 days at -70 °C and thawing at room temperature the fraction released increased from 54.01 ± 0.43% to 57.06 ± 1.05% (n=3, P=0.95). Therefore, an analytical error of approximately 3% in the quantified amount of released doxorubicin can be assumed.

Similarly to the results obtained for the release rates in model media, centrifugation at 15,000 × g led to the presence of higher amounts of doxorubicin in the supernatant as compared to centrifugation at 48,254 × g with a difference of approximately 10% over three different doxorubicin concentrations (10, 50, 100 µg/mL, Figure 2). Therefore, to study the content of free doxorubicin in the blood plasma, the nanoparticles were separated at 48,254 × g. NanoCore-6.4 exhibits a relatively higher initial release rate (burst effect) with the fraction released (60-90%) depending on the initial concentration of nanoparticles. In the case of

NanoCore-7.4, approximately 50-60% of doxorubicin is released into plasma, thus, the release is less dependent on the initial concentration of nanoparticles than in the previous case.

As for the effect of the type of extractant on the determined percentage of free doxorubicin in plasma, when applying a mixture of acetonitrile and DMSO as the extractant, the percentage of free doxorubicin did not change when the nanoparticles were separated by centrifugation at low acceleration (15,000 × g). At higher acceleration of 48,254 × g, a slight but statistically significant reduction (2-3%) was observed. An increase in the recovery of doxorubicin (up to 10-14%) is the most likely explanation.

In summary, for analyzing the release of doxorubicin in human plasma, a mixture of acetonitrile and DMSO (1:1) supplemented with 0.1% formic acid considerably improves the recovery of the compound while an optimal separation is achieved by centrifugation at acceleration of 48,254 × g.

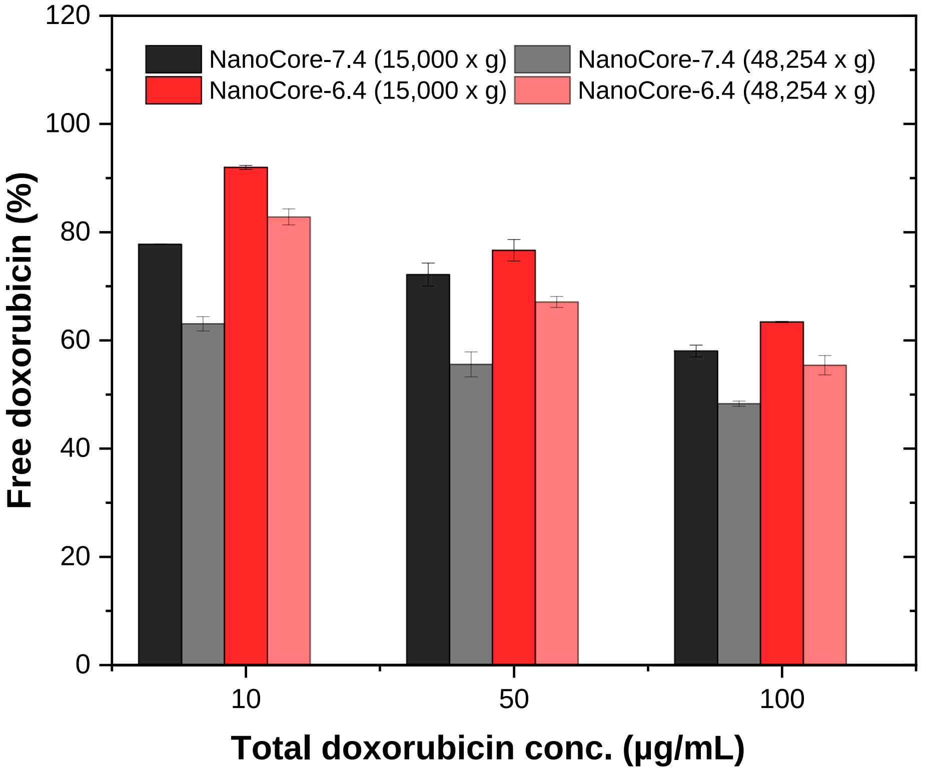

Figure 2: Influence of centrifugation mode on the content of free doxorubicin in blood plasma comparing accelerations of 15,000 × g and 48,254 × g for NanoCore-7.4 (grey) and NanoCore-6.4 (red), n=3.

S5. Quantification of the blood-to-plasma rate ratio ($K_{Blood/Plasma}$) and the erythrocyte-to-plasma rate ratio ($K_{RBC/Plasma}$)

The blood-to-plasma rate ratio ($K_{Blood/Plasma}$) and the erythrocyte-to-plasma rate ratio ($K_{RBC/Plasma}$) of free doxorubicin and the two nanoparticle formulations are presented in Table 5.

Table. 5: Average values of doxorubicin distribution coefficients between blood, plasma, and erythrocytes

Time	Parameter	Doxorubicin concentration (µg/mL)								
		10			50			100		
		Free doxorubicin	NanoCore-7.4	NanoCore-6.4	Free doxorubicin	NanoCore-7.4	NanoCore-6.4	Free doxorubicin	NanoCore-7.4	NanoCore-6.4
**5 min**	$K_{RBC/Plasma}$	0.83	0.72	0.84	0.76	0.69	0.75	0.73	0.68	0.70
	$K_{Blood/Plasma}$	0.93	0.89	0.94	0.90	0.88	0.90	0.89	0.87	0.88
**15** min	$K_{RBC/Plasma}$	2.15	1.42	1.94	2.42	1.28	2.1	2.53	1.42	2.07
	$K_{Blood/Plasma}$	1.46	1.17	1.38	1.57	1.11	1.44	1.61	1.17	1.43
**30** min	$K_{RBC/Plasma}$	2.25	1.50	1.83	2.30	1.52	2.00	2.73	1.34	1.98
	$K_{Blood/Plasma}$	1.50	1.20	1.33	1.52	1.21	1.14	1.69	1.14	1.39

S6. Quantification of doxorubicin in rat plasma

1. Preparation of stock solutions and calibration

The calibration standards were prepared in pooled rat plasma with doxorubicin concentrations ranging from 4-20,000 ng/mL using a doxorubicin stock solution (1 mg/mL), prepared as described previously (S3). To obtain the rat plasma (for plotting the calibration curves), the whole rat blood was collected in K3-EDTA tubes and the red blood cells were immediately separated by centrifugation (1500 x g, +20 °C, 10 min). Plasma was collected and stored at -70 °C until further analysis. The preparation procedure of the calibration stock solutions and the 10X calibration standard solutions of doxorubicin is illustrated in Tables 6 and 7.

Table 6: Preparation of calibration stock solutions

Doxorubicin concentration, ng/mL	**Preparation procedure**
200,000	100 µL [1 mg/mL doxorubicin solution] + 400 µL [ACN:H_2O (1:1) + 0.1% formic acid]
10,000	25 µL [200,000 ng/mL doxorubicin solution] + 475 µL [ACN:H_2O (1:1) + 0.1% formic acid]
1,000	50 µL [10,000 ng/mL doxorubicin solution] + 450 µL [ACN:H_2O (1:1) + 0.1% formic acid]
100	50 µL [1,000 ng/mL doxorubicin solution] + 450 µL [ACN:H_2O (1:1) + 0.1% formic acid]

Before sample preparation, the IS solution (daunorubicin, 2,000 ng/mL) was prepared by adding 475 µL of acetonitrile-water (1:1) supplemented with 0.1% of formic acid to 25 µL of the IS solution (40 µg/mL). The calibration solutions were prepared 3 times from separate aliquots of the stock solution, and the precision and accuracy between the calibration curves were evaluated.

Table 7: Preparation of 10X calibration standard solutions

Calibration standard	Doxorubicin concentration in plasma, ng/mL	Doxorubicin concentration in 10X solution, ng/mL	Doxorubicin concentration in stock solution, ng/mL	Volume of stock solution, µL	Volume of ACN:H_2O (1:1) + 0.1% formic acid, µL
K1	4	40	100	80	120
K2	10	100	100	-	-
K3	20	200	1,000	40	160
K4	40	400	1,000	80	120
K5	100	1,000	1,000	-	-
K6	200	2,000	10,000	40	160
K7	400	4,000	10,000	80	120
K8	700	7,000	10,000	140	60
K9	1,000	10,000	10,000	-	-
K10	4,000	40,000	200,000	40	160
K11	10,000	100,000	200,000	100	100
K12	20,000	200,000	200,000	-	-

2. Sample preparation in rat plasma for calibration

The pooled rat plasma used to prepare the calibration standard solutions were thawed at room temperature and mixed using a vortex mixer. A volume of 20 µL of 10X calibration standard solution of doxorubicin was added to 200 µL of plasma in 1.5 mL tubes and then gently mixed for 2 min. A volume of 20 µL of the IS solution (daunorubicin, 2,000 ng/mL) was added to all samples except for the blank preparations. In the case of blank samples (without analyte), a volume of 20 µL of the blank solvent (acetonitrile-water (1:1) supplemented with 0.1% formic acid) was added. The samples were extracted with 300 µL of cooled (-20 °C) acetonitrile comprising 0.1% of formic acid. After 2 min of shaking at a rate of 1,200 rpm, a volume of 300 µL of DMSO was added followed by shaking at 1,200 rpm for 8 min. The samples were centrifuged for

30 min at 4,000 rpm and a temperature of +4 °C in an Eppendorf 5804R centrifuge (Eppendorf AG, Hamburg, Germany). An aliquot of 300 µL of supernatant was collected for further HPLC analysis.

S7. Non-compartmental analysis of pharmacokinetics

The outcome of the non-compartmental analysis (NCA) is provided in

Table .

Table 8: Pharmacokinetic parameters obtained by non-compartmental analysis for total and free doxorubicin as determined in the pharmacokinetic study.

Formulation	Parameter	Mean	min	max	SD	CV
Doxorubicin (5 mg/kg)	AUC (ng×h/mL)	1300.9	1101.8	1459.9	135.8	10.4
	$AUC_{0\rightarrow 1h}$ (ng×h/mL)	395.3	314.3	546.8	94.2	23.8
	c_{max} (ng/mL)	1265.4	1038.1	1735.8	293.2	23.2
	Cl (mL/h)	662.7	600.4	790.1	68.2	10.3
	V (mL)	13498.2	10483.7	16525.7	2374.4	17.6
NanoCore-7.4_{total} (5 mg/kg)	AUC (ng×h/mL)	4603.8	3294.2	6532.7	1247.1	27.1
	$AUC_{0\rightarrow 1h}$ (ng×h/mL)	3589.5	2329.7	5615.4	1298.5	36.2
	c_{max} (ng/mL)	12441.0	8063.9	19358.4	4276.8	34.4
	Cl (mL/h)	219.5	149.6	286.1	52.5	23.9
	V (mL)	1576.8	547.9	2603.2	789.0	50.0
NanoCore-$7.4_{FreeDox}$ (5 mg/kg)	AUC_{all} (ng×h/mL)	2064.8	1781.1	2476.3	273.9	13.3
	$AUC_{0\rightarrow 1h}$ (ng×h/mL)	1051.6	819.5	1397.9	244.8	23.3
	c_{max} (ng/mL)	3558.2	2590.0	4872.9	945.3	26.6
	Cl (mL/h)	414.4	338.4	455.9	45.6	11.0
	V (mL)	8396.6	5111.2	12427.9	2520.0	30.0
NanoCore-6.4_{Total} (5 mg/kg)	AUC (ng×h/mL)	4434.1	3087.8	5316.0	1017.8	23.0
	$AUC_{0\rightarrow 1h}$ (ng×h/mL)	2999.1	1862.9	3834.2	905.3	30.2
	c_{max} (ng/mL)	10366.8	6948.6	12682.7	2695.4	26.0
	Cl (mL/h)	216.2	169.0	289.1	52.4	24.2
	V (mL)	2506.4	1116.4	4762.4	1242.1	49.6
NanoCore-$6.4_{FreeDox}$ (5 mg/kg)	AUC (ng×h/mL)	2995.3	2402.9	3775.3	574.8	19.2
	$AUC_{0\rightarrow 1h}$ (ng×h/mL)	1647.4	1158.1	2461.6	500.2	30.4
	c_{max} (ng/mL)	5682.8	4216.8	7908.1	1489.5	26.2
	Cl (mL/h)	309.3	239.3	359.7	51.2	16.6
	V (mL)	4224.4	2979.2	6089.0	1262.6	29.9
NanoCore-6.4_{total} (2 mg/kg)	AUC (ng×h/mL)	1937.3	1645.3	2345.7	261.2	13.5
	$AUC_{0\rightarrow 1h}$ (ng×h/mL)	1253.8	1001.5	1667.7	253.7	20.2
	c_{max} (ng/mL)	3065.3	2423.5	4016.7	545.2	17.8
	Cl (mL/h)	142.1	78.3	175.4	36.9	25.9
	V (mL)	7269.9	2625.8	11298.5	2955.2	40.6
NanoCore-$6.4_{FreeDox}$ (2 mg/kg)	AUC_{all} (ng×h/mL)	1368.7	1297.8	1460.5	55.7	4.1
	$AUC_{0\rightarrow 1h}$ (ng×h/mL)	699.5	619.8	784.9	62.3	8.9
	c_{max} (ng/mL)	1886.5	1661.2	2092.7	156.0	8.3
	Cl (mL/h)	191.8	122.7	243.2	40.1	20.9
	V (mL)	9244.7	6712.4	14390.3	2867.9	31.0
